# Anti-inflammatory effects of β-FNA are sex-dependent in a pre-clinical model of LPS-induced inflammation

**DOI:** 10.1186/s12950-023-00328-z

**Published:** 2023-01-25

**Authors:** Stephanie Myers, Kelly McCracken, Daniel J. Buck, J. Thomas Curtis, Randall L. Davis

**Affiliations:** Department of Pharmacology/Physiology, Oklahoma State University Center for Health Sciences, 1111 West 17th Street, Tulsa, OK 74107 USA

**Keywords:** β-funaltrexamine, Neuroinflammation, Neuroprotective, Chemokine, Cytokine, Nuclear factor-κB, Opioid, Peripheral inflammation

## Abstract

**Background:**

Inflammation is present in neurological and peripheral disorders. Thus, targeting inflammation has emerged as a viable option for treating these disorders. Previous work indicated pretreatment with beta-funaltrexamine (β-FNA), a selective mu-opioid receptor (MOR) antagonist, inhibited inflammatory signaling *in vitro* in human astroglial cells, as well as lipopolysaccharide (LPS)-induced neuroinflammation and sickness-like-behavior in mice. This study explores the protective effects of β-FNA when treatment occurs 10 h after LPS administration and is the first-ever investigation of the sex-dependent effects of β-FNA on LPS-induced inflammation in the brain and peripheral tissues, including the intestines.

**Results:**

Male and female C57BL/6J mice were administered LPS followed by treatment with β-FNA-immediately or 10 h post-LPS. Sickness- and anxiety-like behavior were assessed using an open-field test and an elevated-plus-maze test, followed by the collection of whole brain, hippocampus, prefrontal cortex, cerebellum/brain stem, plasma, spleen, liver, large intestine (colon), proximal small intestine, and distal small intestine. Levels of inflammatory chemokines/cytokines (interferon γ-induced-protein, IP-10 (CXCL10); monocyte-chemotactic-protein 1, MCP-1 (CCL2); interleukin-6, IL-6; interleukin-1β, IL-1β; and tumor necrosis factor-alpha, TNF-α) in tissues were measured using an enzyme-linked immunosorbent assay. Western blot analysis was used to assess nuclear factor-kappa B (NF-κB) expression. There were sex-dependent differences in LPS-induced inflammation across brain regions and peripheral tissues. Overall, LPS-induced CXCL10, CCL2, TNF-α, and NF-κB were most effectively downregulated by β-FNA; and β-FNA effects differed across brain regions, peripheral tissues, timing of the dose, and in some instances, in a sex-dependent manner. β-FNA reduced LPS-induced anxiety-like behavior most effectively in female mice.

**Conclusion:**

These findings provide novel insights into the sex-dependent anti-inflammatory effects of β-FNA and advance this agent as a potential therapeutic option for reducing both neuroinflammation an intestinal inflammation.

## Background

Neurological disorders, such as anxiety, stress, and other mood disorders, often involve some level of inflammation [1–6]. Brain regions particularly involved in these neurological disorders include the prefrontal cortex, hippocampus, and cerebellum [7–9]. Indeed, proinflammatory cytokines and chemokines have been implicated as potential pathologically relevant biomarkers or therapeutic targets in psychiatric disorders [10–12]. Inflammation, as evidenced by increased proinflammatory cytokine/chemokine expression, also occurs in the spleen, liver, and/or intestines during certain infections, cirrhosis, inflammatory bowel disease (IBD), Crohn's disease (CD), and ulcerative colitis (UC). [13–16]. Interestingly, anxiety is associated with the onset of new IBD symptoms; and conversely, gastrointestinal symptoms correlate with development of anxiety symptoms [17]. Emerging evidence implicates proinflammatory cytokines/chemokines in the bidirectional interactions between the gut and the central nervous system (CNS) leading to these co-morbidities [18–22].

Proinflammatory cytokines are small secretory proteins that function as intercellular mediators. Microglia and astrocytes are major sources of cytokines in the CNS. Whereas, in the periphery, leukocytes, endothelial cells, and mast cells are key sources of pro-inflammatory cytokines [23, 24]. Chemokines are chemotactic cytokines that are instrumental in regulation of immune functions as well as neuronal functions. Among the most implicated cytokines/chemokines in both neurological disorders and IBD are tumor necrosis factor-α (TNF-α), IL-1β, IL-6, CXCL10, and CCL2. The expression of these factors is regulated in part by intracellular signaling molecules including the p38 mitogen-activated kinase (p38 MAPK) and the transcription factor nuclear factor-kappa B (NF-κB). Thus, inhibition of p38 and/or NF-κB is an effective means of reducing cytokine/chemokine expression.

The increasing evidence that inflammation is instrumental in anxiety and mood disorders, peripheral conditions such as IBD and UC, as well as the co-morbidity of these conditions has led to a strong interest in identifying anti-inflammatory agents as therapeutic options for such disorders. We are interested in the potential benefits of β-funaltrexamine (β-FNA), a selective *mu*-opioid receptor (MOR) antagonist. Interestingly, we previously discovered that β-FNA inhibits inflammatory signaling *in vitro* in human astroglial cells [25–28]. More specifically, β-FNA inhibited cytokine-induced expression of cytokines/chemokines and activation of both nuclear factor-kappa B (NF- κB) and p38 mitogen-activated kinase (p38 MAPK) in human astroglial cells [25, 26, 28–30]. Importantly, these anti-inflammatory actions were not dependent on the actions at the MOR [26].

More recently, we used an *in vivo* approach with adult male C57BL/6J mice to determine β-FNA's ability to inhibit bacterial lipopolysaccharide (LPS)-induced neuroinflammation [27]. We determined that β-FNA administered (i.p.) immediately before LPS injection resulted in reduced neuroinflammation as indicated by suppressed cytokine/chemokine expression in the brain 24 h after treatment [27].

In the present study, the overall working hypothesis is that β-FNA inhibits LPS-induced inflammation and behavioral deficits in mice. More specifically, we expect that β-FNA will inhibit LPS-induced inflammation in female mice given that clinically, the prevalence of anxiety and depression is higher in females than in males [31–33]. In combination, the findings from this study are expected to provide novel insights into the anti-inflammatory actions of β-FNA and foster potential therapeutic options for treating neuroinflammatory and peripheral inflammatory conditions.

## Results

### β-FNA effects on LPS-induced sickness-like behavior

**Fig. 1:**
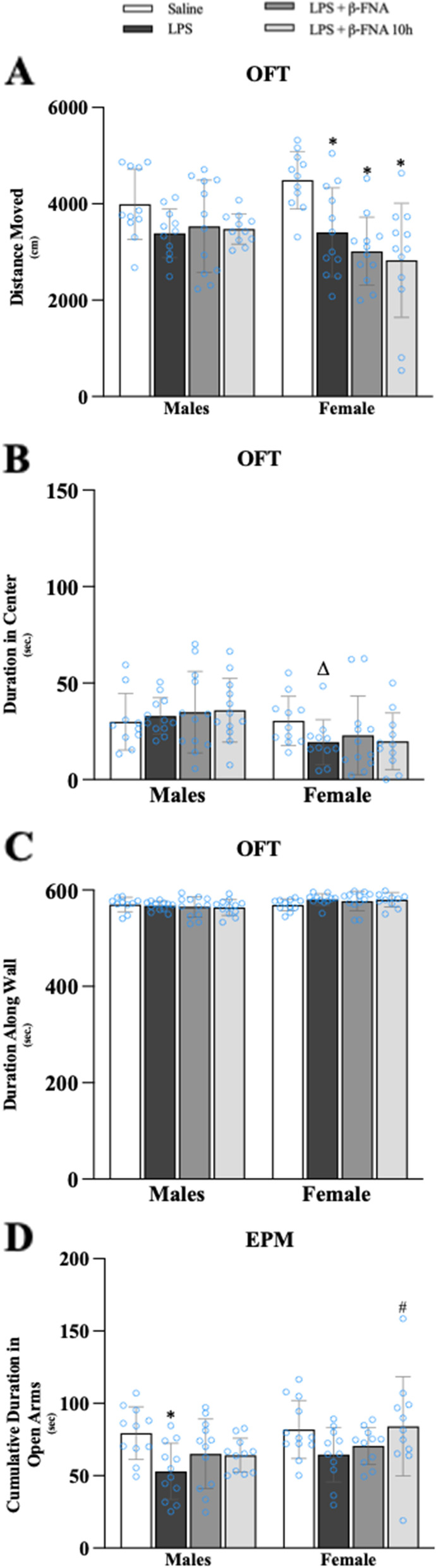
β-FNA effects on LPS-induced behavioral deficits in male and female C57BL/6J mice. Mice (*n* = 11–12/group) were injected (i.p.) with saline (control), LPS (0.83 mg/kg), and LPS followed immediately by β-FNA treatment (50 mg/kg; i.p.; LPS + β-FNA), or LPS followed by β-FNA 10 h post-LPS (LPS + β-FNA 10 h). 24 h post-LPS, assessment of mice was conducted via **A-C** 10 min open-field test (OFT) and **D** 5 min elevated plus maze (EPM). Data are reported as mean ± SEM. Two-way ANOVA indicated a significant main effect of treatment (*p* < 0.0001) on distance moved in the OFT, but no significant effect of sex (*p* = 0.33) and no significant interaction of treatment and sex (*p* = 0.07). Two-way ANOVA indicated a significant main effect of sex (*p* < 0.01) on duration in the center but no main effect of treatment (*p* = 0.85) or interaction (*p* = 0.33). Two-way ANOVA indicated a significant main effect of sex (*p* < 0.01) on duration along the wall but no main effect of treatment (*p* = 0.84) or interaction (*p* = 0.31). Two-way ANOVA revealed significant main effects of sex (*p* < 0.03) and treatment (*p* < 0.01) on time in the open arms in the EPM, but no significant interaction (*p* = 0.52). Pairwise comparisons were assessed using a Fisher’s LSD test; * indicates *p* < 0.05 vs. saline group; # indicates *p* < 0.05 vs. LPS group. Δ indicates *p* < 0.05 vs. males LPS

Two-way ANOVA of the OFT data indicated a significant main effect of treatment (F_3, 84_ = 8.64, *p* < 0.0001) but no main effect of sex (F_1, 84_ = 0.968, *p* = 0.33) or interaction (F_3, 84_ = 2.49, *p* = 0.07) (Fig. 1A). Pairwise comparisons revealed a significant reduction in distance moved by LPS-treated female mice compared to saline-treated female mice (*p* < 0.002). The distance moved by female LPS + β-FNA and LPS + β-FNA 10 h mice was less than that by saline female mice (*p* < 0.0001), but similar to female LPS mice (*p* > 0.05). For male mice, the distance moved by LPS mice was seemingly lower than that of saline male mice, but not to the level of significance (*p* = 0.078). Regardless of the timing, β-FNA treatment did not significantly affect the distance moved by LPS-treated male mice (*p* > 0.05).

Two-way ANOVA of duration in the center for OFT data indicated a significant main effect of sex (F_1, 83_ = 9.73, *p* < 0.01) but no main effect of treatment (F_3, 83_ = 0.26, *p* = 0.85) or interaction (F_3, 83_ = 1.17, *p* = 0.33) (Fig. 1B).

Pairwise comparisons revealed no significant difference in duration in the center by LPS-treated males mice compared to saline-treated male mice (*p* = 0.66). The duration in the center by male LPS + β-FNA (p = 0.46) and LPS + β-FNA 10 h (p = 0.38) mice was also not significantly different to that of saline male mice. For female mice, the duration in the center by LPS mice was not significantly different than that of saline female mice (p = 0.10). Regardless of the timing, β-FNA treatment did not significantly affect the duration in the center by LPS-treated male mice (*p* > 0.05).

Two-way ANOVA of duration along the wall for OFT data indicated a significant main effect of sex (F_1, 83_ = 9.28, *p* < 0.01) but no main effect of treatment (F_3, 83_ = 0.28, *p* = 0.84) or interaction (F_3, 83_ = 1.23, *p* = 0.31) (Fig. 1C). Pairwise comparisons revealed no significant difference in duration along the wall by LPS-treated males mice compared to saline-treated male mice (*p* = 0.67). The duration along the wall by male LPS + β-FNA (*p* = 0.47) and LPS + β-FNA 10 h (*p* = 0.37) mice was also not significantly different to that of saline male mice. For female mice, the duration along the wall by LPS mice was not significantly different than that of saline female mice (*p* = 0.09). Regardless of the timing, β-FNA treatment did not significantly affect the duration along the wall by LPS-treated male mice (*p* > 0.05). Together these OFT data suggest that LPS induced minimal sickness-like behavior, and only in females; and β-FNA did not significantly impact the limited LPS-induced sickness-like behavior.

### β-FNA effects on LPS-induced anxiety-like behavior

Two-way ANOVA of EPM data indicated significant main effects of both treatment (F_3, 83_ = 4.53, *p* < 0.01) and sex (F_1, 83_ = 4.99, *p* < 0.03), but no significant interaction (F_3, 83_ = 0.761, *p* = 0.52) (Fig. 1B). Pairwise comparisons revealed that male LPS mice spent less time in the open arms than did the male saline mice (*p* < 0.004). Whereas male LPS + β-FNA and LPS + β-FNA 10 h mice spent a similar duration in the open arms as did male saline mice (*p* ≥ 0.09). For females, LPS mice spent less time in the open arms compared to saline females; however, not to the level of significance (*p* = 0.051). The LPS + β-FNA female mice spent similar time in the open arms compared to LPS females, whereas the LPS + β-FNA 10 h females spent more time in the open arms than the LPS females (*p* < 0.03). These findings suggest that LPS induces anxiety-like behavior in male mice and marginally in female mice, and β-FNA is protective, particularly in LPS + β-FNA 10 h females.

### Effects of β-FNA on LPS-induced cytokine and chemokine expression in the brain

**Fig. 2:**
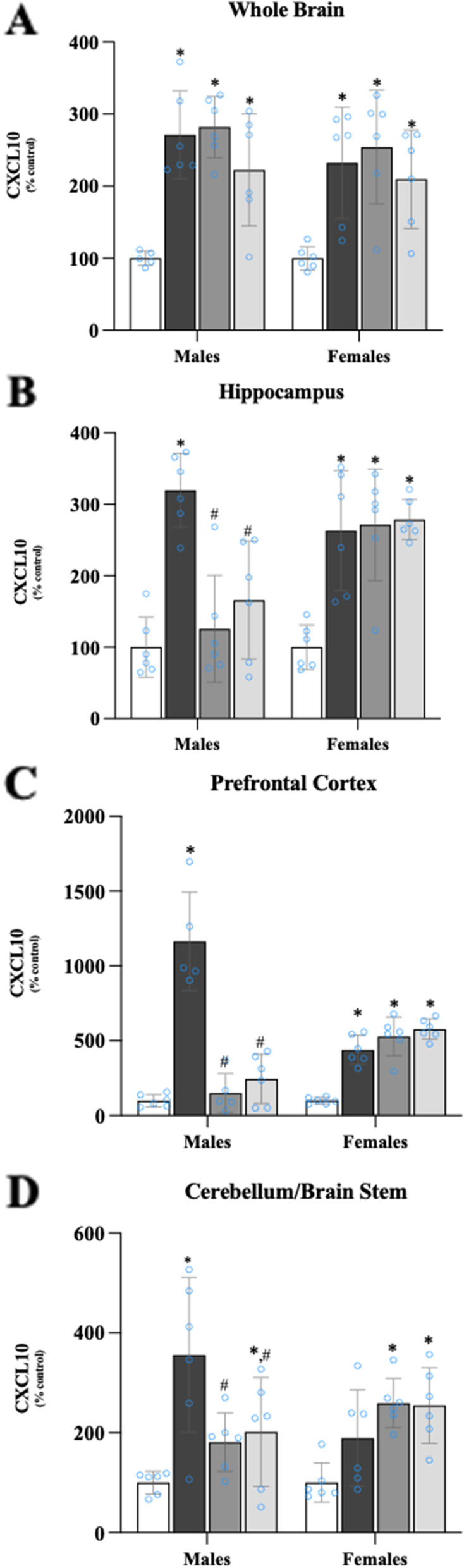
β-FNAs effect on LPS-induced CXCL10 expression in the whole brain and brain regions (hippocampus, prefrontal cortex, and cerebellum/brain stem) of male and female C57BL/6J mice. Mice (*n* = 5–6/group) were injected (i.p.) with saline (control), LPS (0.83 mg/kg), LPS followed immediately by β-FNA treatment (50 mg/kg; i.p.; LPS + β-FNA), or LPS followed by β-FNA 10 h post-LPS (LPS + β-FNA 10 h). 24 h post-LPS, mice were terminated followed by tissue collection. Levels of CXCL10 in whole brain (**A**), hippocampus (**B**), prefrontal cortex (**C**), and cerebellum/brain stem (**D**) were measured by ELISA. Data are reported as mean ± SEM. Two-way ANOVA indicated a significant main effect of treatment (*p* < 0.0001) on CXCL10 levels in the whole brain; but no significant effect of sex (*p* = 0.27), nor a significant interaction (*p* = 0.88). Two-way ANOVA determined CXCL10 in the hippocampus had a significant main effect of sex (*p* < 0.01), treatment (*p* < 0.001), and interactions (*p* < 0.001). In the prefrontal cortex two-way ANOVA determined CXCL10 had a significant main effect of treatment (*p* < 0.0001) and interactions (*p* < 0.0001), but not sex (*p* = 0.94). Two-way ANOVA determined in the cerebellum/brain stem that CXCL10 had a significant main effect of treatment (*p* < 0.0001), and interactions (*p* < 0.01), but not sex (*p* = 0.73). Pairwise comparisons were assessed using a Fisher’s LSD test; * indicates *p* < 0.05 vs. saline group; # indicates *p* < 0.05 vs. LPS group

As determined by two-way ANOVA, there was a significant main effect of treatment on CXCL10 levels in the whole brain (F_3, 39_ = 17.51, *p* < 0.0001), but no significant effect of sex (F_1, 39_ = 1.25, *p* = 0.27) and no interaction (F_3, 39_ = 0.227, *p* = 0.88) (Fig. 2A). Pairwise comparisons showed a significant increase in CXCL10 levels in male and female LPS mice compared to same-sex saline mice (*p* < 0.0001 and *p* < 0.001, respectively). For both males and females, CXCL10 levels in LPS + β-FNA and LPS + β-FNA 10 h mice were significantly higher compared to same-sex saline mice (*p* < 0.01 in all instances).

There were significant main effects of treatment (F_3, 40_ = 18.92, *p* < 0.001) and sex (F_1, 40_ = 7.68, *p* < 0.01) on CXCL10 levels in the hippocampus; and a significant interaction of treatment and sex (F_3, 40_ = 6.80, *p* < 0.001) (Fig. 2B). Pairwise comparisons indicated that CXCL10 levels in the hippocampus of male LPS mice were significantly increased relative to male saline mice (*p* < 0.0001), whereas CXCL10 levels in male LPS + β-FNA and LPS + β-FNA 10 h mice were similar to levels in male saline mice (*p* > 0.05). In the hippocampus of female mice, CXCL10 levels were increased in LPS mice relative to saline mice (*p* < 0.0001); and levels in LPS + β-FNA and LPS + β-FNA 10 h mice were similar to the levels in LPS mice (*p* > 0.05).

Two-way ANOVA revealed a significant main effect of treatment (F_3, 38_ = 45.67, *p* < 0.0001) on CXCL10 levels in the prefrontal cortex, no main effect of sex (F_1, 38_ = 0.0058, *p* = 0.94), and a significant interaction of treatment and sex (F_3, 38_ = 34.12, *p* < 0.0001) (Fig. 2C). Pairwise comparisons indicated that in male mice, CXCL10 levels in the prefrontal cortex of male LPS mice were significantly higher than in saline mice (*p* < 0.0001), whereas CXCL10 levels in male LPS + β-FNA and LPS + β-FNA 10 h mice were similar to levels in male saline mice (*p* > 0.05). In the prefrontal cortex of female LPS mice, CXCL10 levels were significantly higher than in saline mice (*p* < 0.001). In females, CXCL10 levels in the prefrontal cortex of LPS + β-FNA and LPS + β-FNA 10 h mice were similar to the levels in LPS mice (*p* > 0.05).

There was a significant main effect of treatment on CXCL10 expression in the cerebellum/brain stem (F_3, 40_ = 8.88, *p* < 0.0001), but no main effect of sex (F_1, 40_ = 0.123, *p* = 0.73); and there was a significant interaction between main effects (F_3, 40_ = 4.93, *p* < 0.01) (Fig. 2D). CXCL10 levels in the cerebellum/brain stem of male LPS mice were increased compared to male saline mice (*p* < 0.0001) as well as male LPS + β-FNA (*p* < 0.002) and LPS + β-FNA 10 h mice (*p* < 0.0001); levels in LPS + β-FNA were not significantly different from the levels in saline mice (*p* > 0.05). In female mice, CXCL10 levels in the cerebellum/brain stem tended to increase in LPS mice relative to saline mice, but not to the level of significance (*p* = 0.079). The levels of CXCL10 in the cerebellum/brain stem of female LPS + β-FNA and LPS + β-FNA 10 h mice were similar to the levels in female LPS mice (*p* > 0.05). This experiment revealed that treatment with β-FNA downregulated LPS-induced CXCL10 expression in the hippocampus, prefrontal cortex, and cerebellum/brain stem of male mice, but not in those of female mice.

**Fig. 3:**
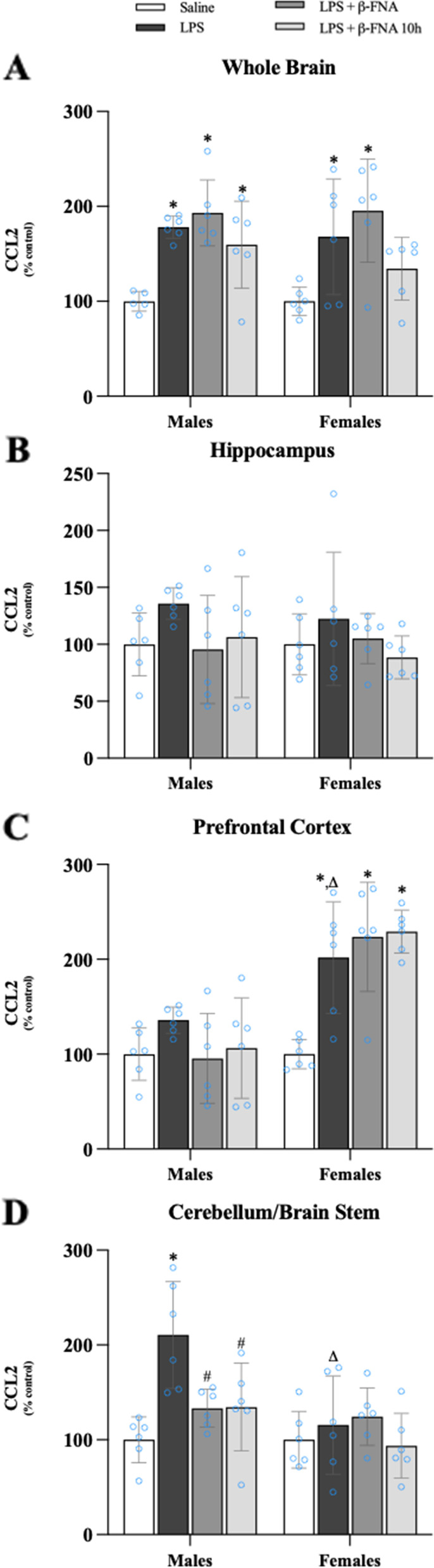
β-FNAs effect on LPS-induced CCL2 expression in the whole brain and brain regions (hippocampus, prefrontal cortex, and cerebellum/brain stem) of male and female C57BL/6J mice. Mice (*n* = 5–6/group) were injected (i.p.) with saline (control), LPS (0.83 mg/kg), LPS followed immediately by β-FNA treatment (50 mg/kg; i.p.; LPS + β-FNA), or LPS followed by β-FNA 10 h post-LPS (LPS + β-FNA 10 h). 24 h post-LPS, mice were terminated followed by tissue collection. Levels of CCL2 of whole brain (**A**), hippocampus (**B**), prefrontal cortex (**C**), and cerebellum/brain stem (**D**) of tissue homogenates were measured by ELISA. Data are reported as mean ± SEM. Two-way ANOVA indicated a significant main effect of treatment (*p* < 0.001) on CCL2 levels in the whole brain; but no significant effect of sex (*p* = 0.47), nor a significant interaction (*p* = 0.81). Two-way ANOVA determined CCL2 in the hippocampus had a no significant main effect of sex (*p* = 0.61), treatment (*p* = 0.14), or interaction (*p* = 0.79). In the prefrontal cortex two-way ANOVA determined CCL2 had a significant main effect of sex (*p* < 0.0001), treatment (*p* < 0.001), and an interaction (*p* < 0.005). Two-way ANOVA determined in the cerebellum/brain stem that CCL2 had a significant main effect of sex (*p* < 0.005), treatment (*p* < 0.005), and an interaction (*p* < 0.02). Pairwise comparisons were assessed using a Fisher’s LSD test; * indicates *p* < 0.05 vs. saline group; # indicates *p* < 0.05 vs. LPS group; Δ indicates *p* < 0.05 vs. males LPS

As determined by two-way ANOVA, there was a significant main effect of treatment on CCL2 expression in the whole brain (F_3, 39_ = 12.69, *p* < 0.001), but no significant effect of sex (F_1, 39_ = 0.545, *p* = 0.47) or an interaction of treatment and sex (F_3, 39_ = 0.316, *p* = 0.81) (Fig. 3A). Pairwise comparisons indicated that CCL2 levels in the whole brain of male and female LPS mice were significantly increased compared to same-sex saline mice (*p* < 0.005 and *p* < 0.002, respectively). In male mice, LPS-induced CCL2 expression in the whole brain was not affected by β-FNA treatment (*p* < 0.05). In female mice, LPS-induced CCL2 expression in the whole brain of LPS + β-FNA mice was similar to the levels in LPS mice (*p* > 0.05), whereas CCL2 levels in LPS + β-FNA 10 h mice were similar to the levels in saline mice (*p* > 0.05) (Fig. 3A).

The levels of CCL2 in the hippocampus were not significantly affected by treatment (F_3, 40_ = 1.95, *p* = 0.14) or sex (F_1, 40_ = 0.259, *p* = 0.61), nor was there an interaction of treatment and sex (F_3, 40_ = 0.344, *p* = 0.79) (Fig. 3B).

In the prefrontal cortex, levels of CCL2 were significantly affected by treatment (F_3, 40_ = 7.69, *p* < 0.001) and sex (F_1, 40_ = 44.48, *p* < 0.0001), with a significant interaction of these main effects (F_3, 40_ = 6.34, *p* < 0.005) (Fig. 3C). There were no significant difference between any of the male treatment groups. However, CCL2 levels in the prefrontal cortex were increased in female LPS mice compared to either female saline mice (*p* = 0.0001) or male LPS mice (*p* < 0.01). The levels of CCL2 in the prefrontal cortex of female LPS + β-FNA and LPS + β-FNA 10 h mice were similar to the levels in female LPS mice (*p* > 0.05).

There were significant main effects of treatment (F_3, 40_ = 5.88, *p* < 0.005) and sex (F_1, 40_ = 10.50, *p* < 0.005) on CCL2 expression in the cerebellum/brain stem, as well as an interaction between treatment and sex (F_3, 40_ = 3.70, *p* < 0.02) (Fig. 3D). Pairwise comparisons indicated a significant increase in CCL2 expression in male LPS mice compared to saline males (*p* < 0.0001) or LPS + β-FNA (*p* < 0.002) and LPS + β-FNA 10 h mice (*p* < 0.002), which were similar to the levels in saline mice. The levels of CCL2 in the cerebellum/brain stem of female mice were not significantly affected by any of the treatments (*p* ≥ 0.28), and the levels in female LPS mice were significantly lower than the levels in male LPS mice (*p* < 0.0001). This experiment indicated that magnitude of LPS-induced CCL2 induction differs among brain regions in a sex-dependent manner (females higher in the prefrontal cortex; and males higher in cerebellum/brain stem). LPS-induced CCL2 expression occurs in the whole brain of male and female mice, and in females, β-FNA at 10 h post LPS is protective. Lastly, β-FNA is protective in the cerebellum/brain stem of males in a male-specific manner.

**Fig. 4:**
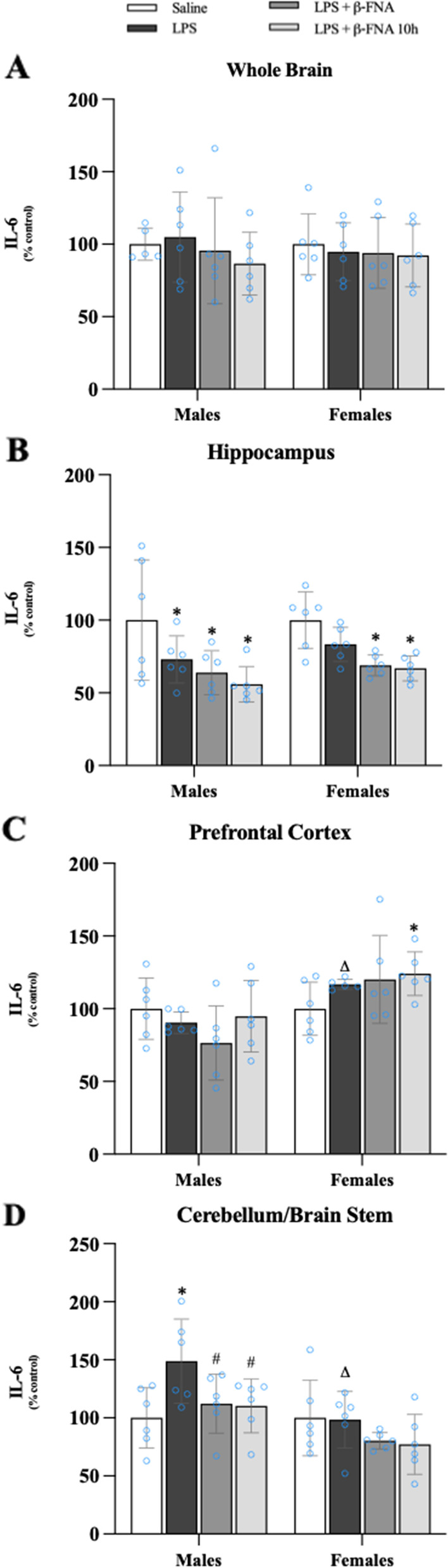
β-FNAs effect on LPS-induced IL-6 expression in the whole brain and brain regions (hippocampus, prefrontal cortex, and cerebellum/brain stem) of male and female C57BL/6J mice. Mice (*n* = 5–6/group) were injected (i.p.) with saline (control), LPS (0.83 mg/kg), LPS followed immediately by β-FNA treatment (50 mg/kg; i.p.; LPS + β-FNA), or LPS followed by β-FNA 10 h post-LPS (LPS + β-FNA 10 h). 24 h post-LPS, mice were terminated followed by tissue collection. Levels of IL-6 in whole brain (**A**), hippocampus (**B**), prefrontal cortex (**C**), and cerebellum/brain stem (**D**) of tissue homogenates were measured by ELISA. Data are reported as mean ± SEM. Two-way ANOVA indicated no significant main effect of treatment (*p* = 0.70), sex (*p* = 0.84), or interaction (*p* = 0.89) on IL-6 levels in the whole brain. Two-way ANOVA determined IL-6 in the hippocampus showed significant main effects of treatment (*p* < 0.0001), but not sex (*p* = 0.25), or interaction (*p* = 0.89). In the prefrontal cortex two-way ANOVA determined IL-6 showed significant main effects of sex (*p* < 0.001), but not treatment (*p* = 0.56), or interaction (*p* = 0.083). Two-way ANOVA determined in the cerebellum/brain stem that IL-6 showed significant main effects of sex (*p* < 0.001) and treatment (*p* < *p* < 0.04) but not interaction (*p* = 0.14). Pairwise comparisons were assessed using a Fisher’s LSD test; * indicates *p* < 0.05 vs. saline group; # indicates *p* < 0.05 vs. LPS group; Δ indicates *p* < 0.05 vs. males LPS

The levels of IL-6 in the whole brain were not significantly affected by treatment (F_3, 39_ = 0.484, *p* = 0.70) or sex (F_1, 39_ = 0.043, *p* = 0.84), nor was there an interaction of treatment and sex (F_3, 39_ = 0.208, *p* = 0.89) (Fig. 4A). In the hippocampus the levels of IL-6 were significantly affected by treatment (F_3, 40_ = 9.42, *p* < 0.0001) but not by sex (F_1, 40_ = 1.39, *p* = 0.25); and there was no interaction (F_3, 40_ = 0.208, *p* = 0.89) (Fig. 4B). The levels of IL-6 in the hippocampus of male LPS mice were significantly lower than in male saline mice (*p* < 0.05). Male LPS + β-FNA and LPS + β-FNA 10 h mice also had decreased IL-6 levels in the hippocampus compared to male saline mice (*p* < 0.005), yet the IL-6 levels were similar to those in LPS mice (*p* > 0.05). In the hippocampus of female mice, IL-6 levels in LPS-treated mice were similar to the levels in saline mice (*p* = 0.15), whereas the levels of IL-6 in LPS + β-FNA and LPS + β-FNA 10 h mice were significantly lower than the levels in saline mice (*p* < 0.01).

There was a significant main effect of sex (F_1, 39_ = 17.41, *p* < 0.001) on IL-6 levels in the prefrontal cortex, but no effect of treatment (F_3, 39_ = 0.701, *p* = 0.56) and no significant interaction (F_3, 39_ = 2.39, *p* = 0.083) (Fig. 4C). The levels of IL-6 in the prefrontal cortex of mice were not significantly affected by any of the treatments (*p* > 0.05). In females, the levels of IL-6 in the prefrontal cortex of LPS mice were significantly greater than the levels in male LPS mice (*p* < 0.04). The levels of IL-6 in the prefrontal cortex of female saline mice were similar to the levels in both LPS (*p* = 0.18) and LPS + β-FNA (*p* = 0.10) mice, yet less than the levels in LPS + β-FNA 10 h mice (*p* < 0.05).

There were significant main effects of treatment (F_3, 40_ = 3.25, *p* < 0.04) and sex (F_1, 40_ = 14.47, *p* < 0.001) on IL-6 expression in the cerebellum/brain stem, but no significant interaction of these effects (F_3, 40_ = 1.918, *p* = 0.14) (Fig. 4D). Pairwise comparisons revealed that IL-6 levels in the cerebellum/brain stem of male LPS mice were higher than the levels in in male saline mice (*p* < 0.01). The levels of IL-6 in LPS + β-FNA and LPS + β-FNA 10 h mice were significantly less than in LPS mice (*p* < 0.03 and *p* < 0.02, respectively) and similar to the levels in male saline mice (*p* > 0.05). In females, there were no significant differences between any treatment groups in terms of IL-6 expression in the cerebellum/brain stem; however, the levels of IL-6 in female LPS mice were significantly lower compared to the levels in male LPS mice (*p* < 0.002).

**Fig. 5:**
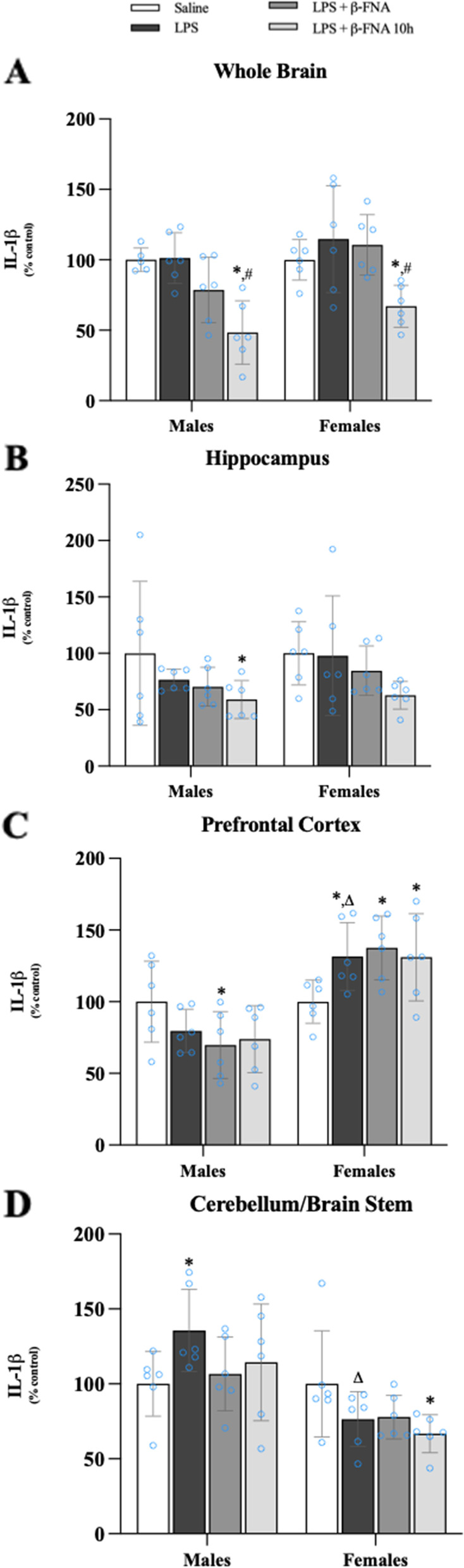
β-FNAs effect on LPS-induced IL-1β expression in the whole brain and brain regions (hippocampus, prefrontal cortex, and cerebellum/brain stem) of male and female C57BL/6J mice. Mice (*n* = 5–6/group) were injected (i.p.) with saline (control), LPS (0.83 mg/kg), LPS followed immediately by β-FNA treatment (50 mg/kg; i.p.; LPS + β-FNA), or LPS followed by β-FNA 10 h post-LPS (LPS + β-FNA 10 h). 24 h post-LPS, mice were terminated followed by tissue collection. Levels of IL-1β of whole brain (**A**), hippocampus (**B**), prefrontal cortex (**C**), and cerebellum/brain stem (**D**) of tissue homogenates were measured by ELISA. Data are reported as mean ± SEM. Two-way ANOVA indicated significant main effects of treatment (*p* < 0.001) and sex (*p* < 0.02) on IL-1β levels in the whole brain, but no significant effect of interaction (*p* = 0.39). Two-way ANOVA determined IL-1β in the hippocampus had a significant main effect of treatment (*p* < 0.05), but not sex (*p* = 0.32) or interaction (*p* = 0.86). In the prefrontal cortex two-way ANOVA determined IL-1β had a significant main effect of sex (*p* < 0.0001) and interaction (*p* < 0.01) but not treatment (*p* = 0.95). Two-way ANOVA determined in the cerebellum/brain stem that IL-1β had a significant main effect of sex (*p* < 0.001) and interaction (*p* = 0.04), but not treatment (*p* = 0.43). Pairwise comparisons were assessed using a Fisher’s LSD test; * indicates *p* < 0.05 vs. saline group; # indicates *p* < 0.05 vs. LPS group; Δ indicates *p* < 0.05 vs. males LPS

Two-way ANOVA indicated that IL-1β levels in the whole brain were significantly affected by treatment (F_3, 39_ = 12.25, *p* < 0.001) and sex (F_1, 39_ = 6.23, *p* < 0.02), but there was not a significant interaction (F_3, 39_ = 1.04, *p* = 0.39) (Fig. 5A). Pairwise comparisons determined that in both male and female mice, IL-1β levels in the whole brain were similar between LPS and saline mice of the same-sex (*p* = 0.93 and *p* = 0.25 for males and females, respectively). Likewise, male and female LPS + β-FNA mice had similar IL-1β levels in the whole brain compared to saline counterparts (*p* = 0.11 and *p* = 0.41, respectively). However, in both males and females, LPS + β-FNA 10 h mice expressed significantly less IL-1β in the whole brain compared to either saline (*p* < 0.001 and *p* < 0.02, respectively) or LPS (both *p* < 0.001) mice.

The IL-1β levels in the hippocampus were significantly affected by treatment (F_3, 40_ = 2.90, *p* < 0.05) but not by sex (F_1, 40_ = 1.02, *p* = 0.32) and there was no significant interaction of treatment and sex (F_3, 40_ = 0.252, *p* = 0.86) (Fig. 5B). The only significant difference detected by pairwise comparisons was IL-1β expression in the hippocampus of male LPS + β-FNA 10 h mice was lower than in male saline mice (*p* < 0.05).

In the prefrontal cortex, IL-1β levels were significantly affected by sex (F_1, 40_ = 43.42, *p* < 0.0001), but not by treatment (F_3, 40_ = 0.121, *p* = 0.95) (Fig. 5C); and there was a significant interaction of treatment and sex (F_3, 40_ = 5.07, *p* < 0.01). In males, IL-1β expression in the hippocampus was significantly lower in LPS + β-FNA mice compared to saline mice (*p* < 0.03), whereas LPS and LPS + β-FNA 10 h mice had IL-1β levels in the prefrontal cortex similar to those in saline mice (*p* = 0.14 and *p* = 0.06, respectively). In females, prefrontal cortex IL-1β expression was increased in LPS mice compared to female saline mice (*p* < 0.03) as well as male LPS mice (*p* < 0.001). Levels of IL-1β in the prefrontal cortex of female LPS + β-FNA and LPS + β-FNA 10 h mice were similar to LPS mice (*p* > 0.05) and greater than saline mice (*p* < 0.01 and *p* < 0.05, respectively).

In the cerebellum/brain stem, IL-1β levels were significantly affected by sex (F_1, 40_ = 20.75, *p* < 0.001) and a significant sex × treatment interaction (F_3, 40_ = 3.02, *p* = 0.04) but no main effect of treatment (F_3, 40_ = 0.933, *p* = 0.43) (Fig. 5D). In males, the levels of IL-1β in the cerebellum/brain stem were increased in LPS mice compared to saline mice (*p* < 0.03), whereas the levels in LPS + β-FNA and LPS + β-FNA 10 h mice were similar to the levels in saline mice (*p* = 0.66 and *p* = 0.34, respectively). In females, IL-1β levels in the cerebellum/brain stem were only significantly affected in LPS + β-FNA 10 h mice, as indicated by reduced expression compared to saline mice (*p* < 0.05). Also, LPS-induced IL-1β expression in the cerebellum/brain stem was less in females than in males (*p* < 0.05).

We conclude from this experiment that LPS-induced IL-1β expression was limited in the brain with upregulation only in the prefrontal cortex of females and in the cerebellum/brain stem of males.

**Fig. 6:**
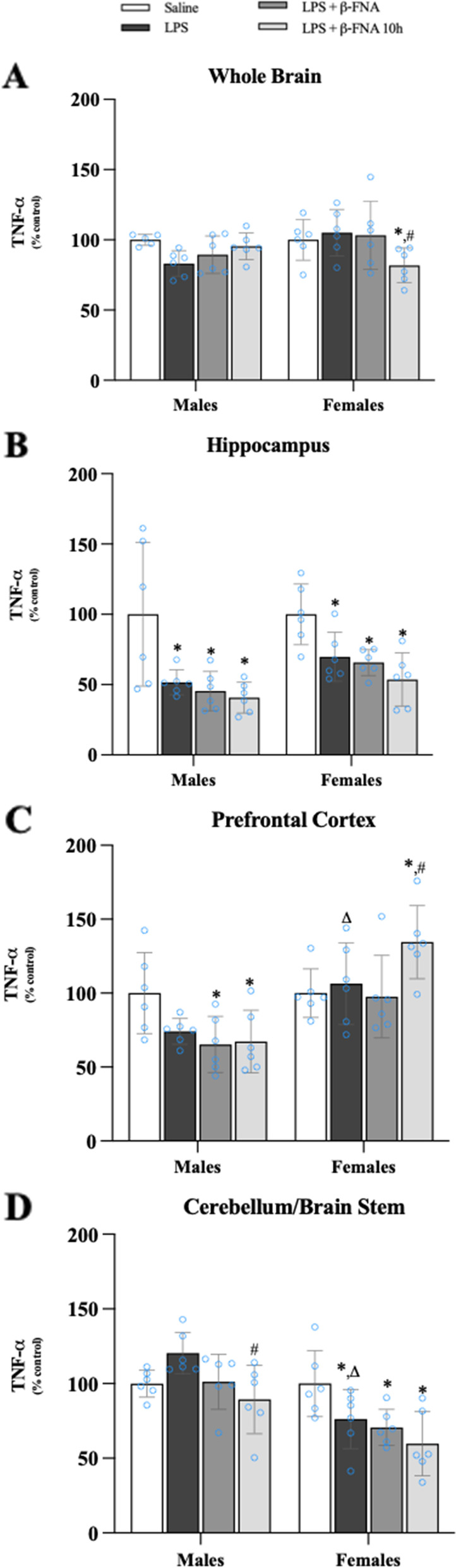
β-FNAs effect on LPS-induced TNF-α expression in the whole brain and brain regions (hippocampus, prefrontal cortex, and cerebellum/brain stem) of male and female C57BL/6J mice. Mice (*n* = 5–6/group) were injected (i.p.) with saline (control), LPS (0.83 mg/kg), LPS followed immediately by β-FNA treatment (50 mg/kg; i.p.; LPS + β-FNA), or LPS followed by β-FNA 10 h post-LPS (LPS + β-FNA 10 h). 24 h post-LPS, mice were terminated followed by tissue collection. Levels of TNF-α of whole brain (**A**), hippocampus (**B**), prefrontal cortex (**C**), and cerebellum/brain stem (**D**) of tissue homogenates were measured by ELISA. Data are reported as mean ± SEM. Two-way ANOVA indicated no significant main effect of treatment (*p* = 0.29) or sex (*p* = 0.19), but there was a significant interaction (*p* < 0.03) on TNF-α levels in the whole brain. Two-way ANOVA determined TNF-α in the hippocampus had a significant main effect of treatment (*p* < 0.001) but not sex (*p* = 0.06) and interaction (*p* = 0.70). In the prefrontal cortex two-way ANOVA determined TNF-α had a significant main effect of sex (*p* < 0.0001) and treatment (*p* < 0.01) but not interaction (*p* = 0.13). Two-way ANOVA determined in the cerebellum/brain stem that TNF-α had a significant main effect of sex (*p* < 0.0001), treatment (*p* < 0.01), and interaction (*p* < 0.04). Pairwise comparisons were assessed using a Fisher’s LSD test; * indicates *p* < 0.05 vs. saline group; # indicates *p* < 0.05 vs. LPS group; Δ indicates *p* < 0.05 vs. males LPS

Neither treatment nor sex significantly affected TNF-α levels in the whole brain (F_3, 39_ = 1.28, *p* = 0.29; F_1, 39_ = 1.76, *p* = 0.19, respectively), yet there was a significant interaction (F_3, 39_ = 3.58, *p* < 0.03) (Fig. 6A). In males, TNF-α levels in the whole brain were similar among all treatment groups (*p* > 0.05). In females, pairwise comparisons revealed that LPS + β-FNA 10 h mice expressed less TNF-α than did either saline (*p* < 0.04) or LPS (*p* < 0.01) mice. LPS-induced TNF-α expression in the whole brain was significantly greater in females than in males (*p* < 0.02).

The expression of TNF-α in the hippocampus was significantly affected by treatment (F_3, 40_ = 12.52, *p* < 0.001), but there was no main effect of sex (F_1, 40_ = 3.75, *p* = 0.06), and no significant interaction of the main effects (F_3, 40_ = 0.472, *p* = 0.70) (Fig. 6B). Levels of TNF-α in the hippocampus of male mice were similar among LPS, LPS + β-FNA, and LPS + β-FNA 10 h mice (*p* > 0.05) and TNF-α levels in all three groups were significantly lower than in saline mice (*p* < 0.0007). Similarly, in females, TNF-α expression in the hippocampus did not differ significantly among LPS, LPS + β-FNA, and LPS + β-FNA 10 h mice (*p* > 0.05), and TNF-α levels in these three groups were significantly lower than in the saline group (*p* < 0.03).

In the prefrontal cortex, TNF-α levels were significantly affected by treatment (F_3, 40_ = 4.45, *p* < 0.01) and sex (F_1, 40_ = 25.7, *p* < 0.0001) but there was not a significant interaction (F_3, 40_ = 1.99, *p* = 0.13) (Fig. 6C). Male LPS mice tended to express less TNF-α in the prefrontal cortex than did male saline mice, but not to the level of significance (*p* = 0.054). Male LPS + β-FNA and LPS + β-FNA 10 h mice had significantly lower levels of TNF-α in the prefrontal cortex compared to male saline mice (both *p* < 0.02). Levels of TNF-α in the prefrontal cortex of female LPS mice were greater than the levels in male LPS mice (*p* < 0.02), yet they were similar to the levels in female saline mice (*p* = 0.62) and female LPS + β-FNA mice (*p* = 0.86). However, female LPS + β-FNA 10 h mice expressed significantly more TNF-α in the prefrontal cortex than did either female saline mice (*p* < 0.02) or female LPS mice (*p* < 0.04).

Two-way ANOVA revealed that there were significant main effects of treatment (F_3, 40_ = 5.11, *p* < 0.01) and sex (F_1, 40_ = 24.77, *p* < 0.0001), as well as a significant interaction (F_3, 40_ = 3.16, *p* < 0.04) on TNF-α levels in the cerebellum/brain stem (Fig. 6D). In males, LPS mice appeared to have higher levels of TNF-α in the cerebellum/brain stem than did saline mice, but it was not a significant increase (*p* = 0.06). The only significant difference in cerebellum/brain stem TNF-α expression among males was a decrease in LPS + β-FNA 10 h mice compared to LPS mice (*p* < 0.01). In females, TNF-α expression in the cerebellum/brain stem of LPS, LPS + β-FNA, and LPS + β-FNA 10 h mice were significantly lower than the levels in saline mice (*p* < 0.03, *p* < 0.01, and *p* < 0.001, respectively). Also, LPS-induced TNF-α expression in the cerebellum/brain stem was less in females than in males (*p* < 0.0002).

From this experiment, we concluded that LPS did not upregulate TNF-α expression in the brain. Except in the prefrontal cortex, LPS in combination with β-FNA resulted in a downregulation of TNF-α expression, particularly in LPS + β-FNA 10 h mice.

### Effects of β-FNA on LPS-induced NF-κB-p65expression in brain

**Fig. 7:**
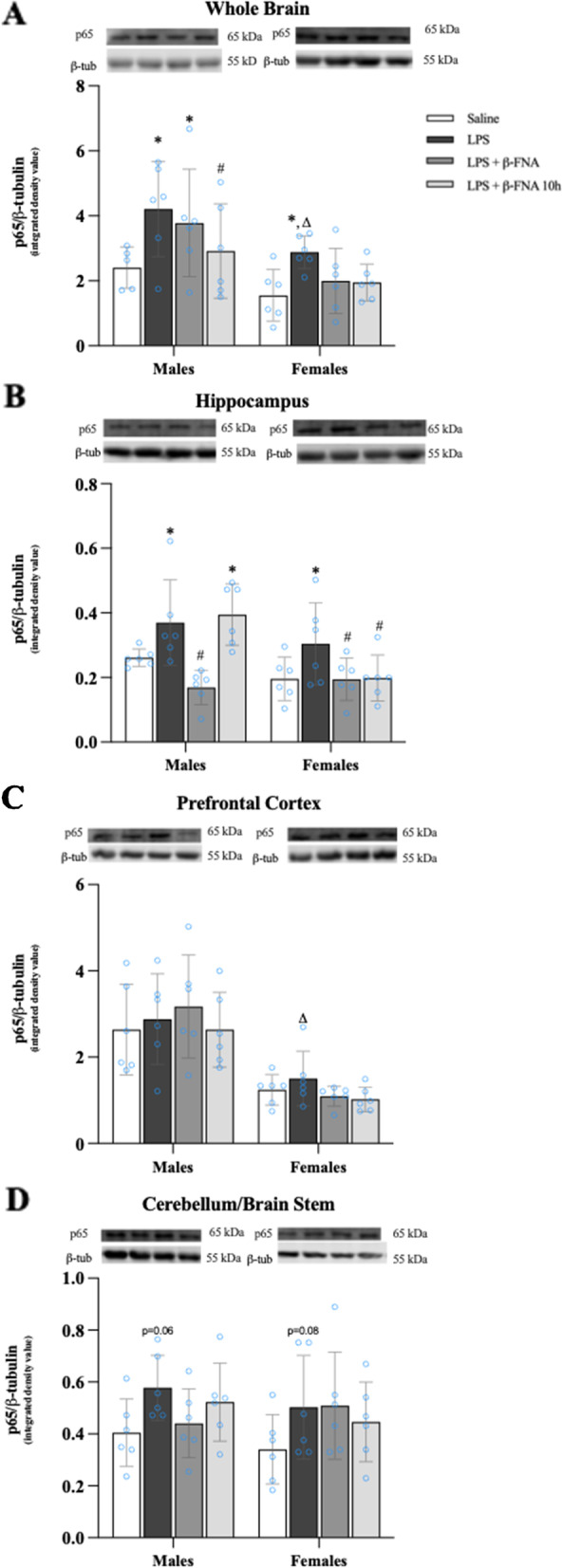
β-FNAs effect on LPS-induced NF-κB-p65 expression in the whole brain and brain regions (hippocampus, prefrontal cortex, and cerebellum/brain stem) of male and female C57BL/6J mice. Mice (*n* = 5–6/group) were injected (i.p.) with saline (control), LPS (0.83 mg/kg), LPS followed immediately by β-FNA treatment (50 mg/kg; i.p.; LPS + β-FNA), or LPS followed by β-FNA 10 h post-LPS (LPS + β-FNA 10 h). 24 h post-LPS, mice were terminated followed by tissue collection. Levels of NF-κB-p65 of whole brain (**A**), hippocampus (**B**), prefrontal cortex (**C**), and cerebellum/brain stem (**D**) of tissue homogenates were measured by western blot analysis (representative western blots are shown above each area analyzed). Data are reported as mean ± SEM. Two-way ANOVA indicated a significant main effect of treatment (*p* < 0.02) and sex (*p* < 0.0001) on levels of NF-κB-p65 in the whole brain; but no significant effect of interaction (*p* = 0.74). Two-way ANOVA determined NF-κB-p65 in the hippocampus had a significant main effect of sex (*p* < 0.01), treatment (*p* < 0.001), and interaction (*p* < 0.03). In the prefrontal cortex two-way ANOVA determined levels of NF-κB-p65 had a significant main effect of sex (*p* < 0.0001), but not treatment (*p* = 0.66) or interaction (*p* = 0.68). Two-way ANOVA determined in the cerebellum/brain stem that NF-κB-p65 had a no significant main effect of sex (*p* = 0.42), treatment (*p* = 0.08), or interaction (*p* = 0.62). Pairwise comparisons were assessed using a Fisher’s LSD test; * indicates *p* < 0.05 vs. saline group; # indicates *p* < 0.05 vs. LPS group; Δ indicates *p* < 0.05 vs. males LPS

Two-way ANOVA indicated significant main effects of treatment (F_3, 39_ = 4.21, *p* < 0.02) and sex (F_1, 40_ = 24.77, *p* < 0.0001) on the levels of NF-κB-p65 in the whole brain, but there was not a significant interaction of these main effects (F_3, 39_ = 0.423, *p* = 0.74) (Fig. 7A). Pairwise comparisons revealed that NF-κB-p65 levels were significantly higher in the brain of male LPS mice compared to male saline mice (*p* < 0.02). NF-κB-p65 levels in the brain of male LPS + β-FNA mice were similar to the levels in male LPS mice (*p* > 0.05), yet the levels in male LPS + β-FNA 10 h mice were similar to the levels in male saline mice (*p* = 0.45). In females, NF-κB-p65 expression in the brain was significantly increased in LPS mice compared to saline mice (*p* < 0.05), whereas the expression in LPS + β-FNA and LPS + β-FNA 10 h mice was similar to that in saline mice (*p* = 0.50 and *p* = 0.54, respectively). LPS-induced NF-κB-p65 expression was significantly lower in females compared to males (*p* < 0.05).

There were significant main effects of treatment (F_3, 40_ = 7.585 *p* < 0.001) and sex (F_1, 40_ = 9.02 *p* < 0.01) on NF-κB-p65 levels in the hippocampus, and a significant interaction (F_3, 40_ = 3.304 *p* < 0.03) (Fig. 7B). Pairwise comparisons indicated that NF-κB-p65 levels in the hippocampus of males were greater in LPS and LPS + β-FNA 10 h mice compared to saline mice (*p* < 0.04 and *p* < 0.02, respectively); while the levels in LPS + β-FNA mice were significantly less than the levels in LPS mice (*p* < 0.001). In females, NF-κB-p65 expression in the hippocampus was significantly upregulated in LPS mice relative to saline mice (*p* < 0.04), whereas levels in LPS + β-FNA and LPS + β-FNA 10 h mice were downregulated (*p* < 0.05) relative to those observed in saline mice.

The levels of NF-κB-p65 in the prefrontal cortex were impacted by a main effect of sex (F_1, 40_ = 49.55 *p* < 0.0001) but not treatment (F_3, 40_ = 0.538 *p* = 0.66), nor was there a significant interaction (F_3, 40_ = 0.508 *p* = 0.68) (Fig. 7C). Pairwise comparisons did not reveal any significant differences in NF-κB-p65 levels in the prefrontal cortex of male mice (*p* ≥ 0.25 Likewise, in female mice, no significant differences were detected in prefrontal cortex NF-κB-p65 levels among treatment groups (*p* ≥ 0.57). However, LPS-induced NF-κB-p65 expression was significantly less in the prefrontal cortex of female mice compared to that in male mice (*p* < 0.01).

NF-κB-p65 levels in the cerebellum/brain stem were not significantly affected treatment (F_3, 40_ = 2.38 *p* = 0.08), sex (F_1, 40_ = 0.655 *p* = 0.42) nor was there a significant interaction (F_3, 40_ = 0.60 *p* = 0.62) (Fig. 7D). Pairwise comparisons revealed no significant differences, however, both male and female LPS mice tended to have increased NF-κB-p65 levels in the cerebellum/brain stem compared to respective saline mice (*p* = 0.06 and *p* = 0.08, respectively). Based on these findings, we conclude that the inhibitory effect of β-FNA on LPS-induced NF-κB-p65 expression in the hippocampus differs in a sex-dependent manner. More specifically, β-FNA treatment immediately post-LPS is most effective in males, whereas, in females, either immediate or delayed (10 h) β-FNA treatment effectively reduces hippocampal NF-κB-p65 expression.

### Effects of β-FNA on LPS-induced cytokine and chemokine levels in plasma

**Fig. 8:**
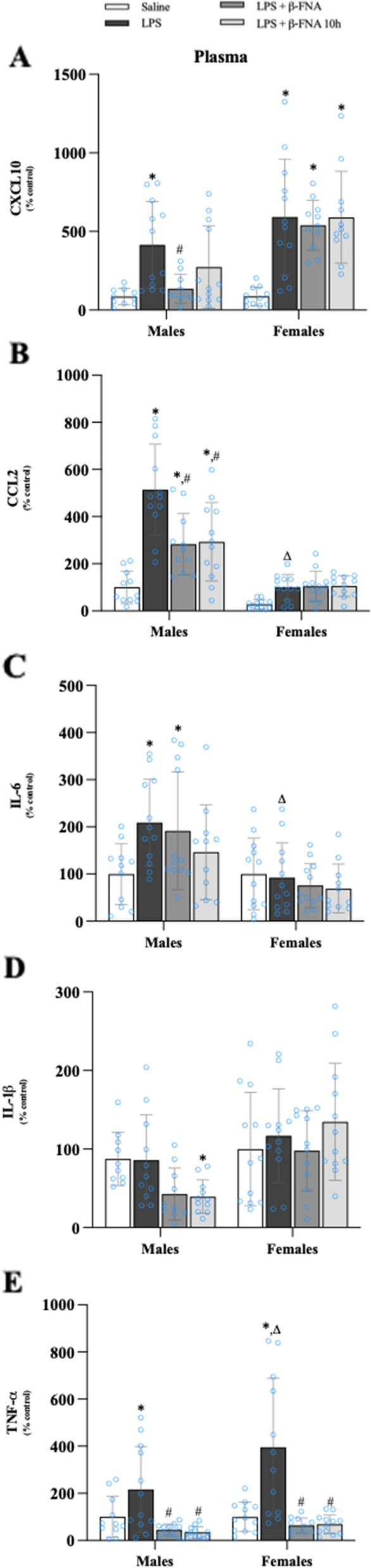
β-FNAs effect on LPS-induced CXCL10, CCL2, IL-6, IL-1β, and TNF-α expression in the plasma of male and female C57BL/6J mice. Mice (*n* = 11–12/group) were injected (i.p.) with saline (control), LPS (0.83 mg/kg), LPS followed immediately by β-FNA treatment (50 mg/kg; i.p.; LPS + β-FNA), or LPS followed by β-FNA 10 h post-LPS (LPS + β-FNA 10 h). 24 h post-LPS, mice were terminated followed by plasma collection. Levels of CXCL10 (**A**), CCL2 (**B**), IL-6 (**C**), IL-1β (**D**), and TNF-α (**E**) of plasma were measured by ELISA. Data are reported as mean ± SEM. Two-way ANOVA indicated a significant main effect of treatment (*p* < 0.0001), sex (*p* < 0.0001), and interaction (*p* < 0.04) on CXCL10 levels in the plasma. Two-way ANOVA determined CCL2 had a significant main effect of sex (*p* < 0.0001), treatment (*p* < 0.0001), and interaction (*p* < 0.0001). Two-way ANOVA determined IL-6 had a significant main effect of sex (*p* < 0.0001) but not treatment (*p* = 0.14) and interaction (*p* = 0.06). Two-way ANOVA determined IL-1β had a significant main effect of sex (*p* < 0.0001) but not treatment (*p* = 0.28) or interaction (*p* = 0.07). Two-way ANOVA determined TNF-α had a significant main effect of sex (*p* < 0.04) and treatment (*p* < 0.0001) but not interaction (*p* = 0.08). Pairwise comparisons were assessed using a Fisher’s LSD test; * indicates *p* < 0.05 vs. saline group; # indicates *p* < 0.05 vs. LPS group; Δ indicates *p* < 0.05 vs. males LPS

Two-way ANOVA indicated that there were significant main effects of treatment (F_3, 81_ = 13.60, *p* < 0.0001), sex (F_1, 81_ = 20.94, *p* < 0.0001), and a significant interaction (F_3, 81_ = 3.07, *p* < 0.04) of treatment and sex on CXCL10 levels in the plasma (Fig. 8A). Pairwise comparisons revealed a significant increase in CXCL10 in the plasma of male LPS mice compared to male saline mice (*p* < 0.002), whereas the levels in LPS + β-FNA and LPS + β-FNA 10 h mice were not significantly different from those of saline males (*p* > 0.05). In females, the levels of CXCL10 in the plasma were significantly increased in LPS, LPS + β-FNA, and LPS + β-FNA 10 h mice versus saline mice (all *p* < 0.0001) with no significant differences among these three treatment groups.

Plasma CCL2 levels were significantly affected by treatment (F_3, 86_ = 19.28, *p* < 0.0001) and sex (F_1, 86_ = 88.56, *p* < 0.0001), with a significant interaction (F_3, 86_ = 10.19, *p* < 0.0001) (Fig. 8B). Male LPS mice had significantly higher plasma CCL2 levels than did male saline mice (*p* < 0.0001). The levels of plasma CCL2 were significantly lower in male LPS + β-FNA and LPS + β-FNA 10 h mice compared to male LPS mice (both *p* < 0.0001). There were no significant differences in plasma CCL2 levels among the groups of female mice (*p* ≥ 0.09). However, LPS upregulation of plasma CCL2 was significantly lower in female mice compared to male mice (*p* < 0.0001).

IL-6 levels in the plasma were significantly impacted by sex (F_1, 85_ = 20.31, *p* < 0.0001) but not by treatment (F_3, 85_ = 1.85, *p* = 0.14); nor was there an interaction of treatment and sex (F_3, 35_ = 2.55, *p* = 0.06) (Fig. 8C). Pairwise comparisons indicated that plasma IL-6 levels were significantly increased in male LPS and LPS + β-FNA mice relative to male saline males (*p* < 0.01), whereas the levels in LPS + β-FNA 10 h mice were not significantly greater than in saline mice (*p* = 0.19). There were no significant differences in plasma IL-6 levels among the groups of female mice (*p* ≥ 0.40), yet LPS upregulation of plasma IL-6 was significantly lower in female mice than in male mice (*p* < 0.001).

There was a significant main effect of sex (F_1, 82_ = 17.63, *p* < 0.0001) on IL-1β levels in the plasma, but not a significant effect of treatment (F_3, 82_ = 1.30, *p* = 0.28) and there was no interaction (F_3, 82_ = 2.4, *p* = 0.07) (Fig. 8D). The only significant difference among the male groups was a lower level of plasma IL-1β in LPS + β-FNA 10 h mice compared to saline mice (*p* < 0.05); and there were no significant differences among the female groups.

Plasma TNF-α levels were significantly affected by treatment (F_3, 86_ = 20.30, *p* < 0.0001) and sex (F_1, 86_ = 4.58, *p* < 0.04), but there was not a significant interaction (F_3, 86_ = 2.31, *p* = 0.08) (Fig. 8E). Male LPS mice had significantly higher levels of plasma TNF-α than did saline males (*p* < 0.04), LPS + β-FNA (*p* < 0.002) or LPS + β-FNA 10 h (*p* < 0.002). Likewise, in female mice, plasma TNF-α levels were significantly upregulated in LPS mice compared to saline mice to either the LPS + β-FNA or LPS + β-FNA 10 h mice. Also, LPS-induced elevation in plasma TNF-α level was significantly more pronounced in females than in males (*p* < 0.002).

These findings indicate that LPS-mediated increases in plasma CXCL10, CCL2, and TNF-α levels are most responsive to the inhibitory effects of β-FNA. Also, the inhibitory actions of β-FNA on CXCL10 and CCL2 are more apparent in males.

### Effects of β-FNA on LPS-induced cytokine and chemokine levels in the spleen

**Fig. 9:**
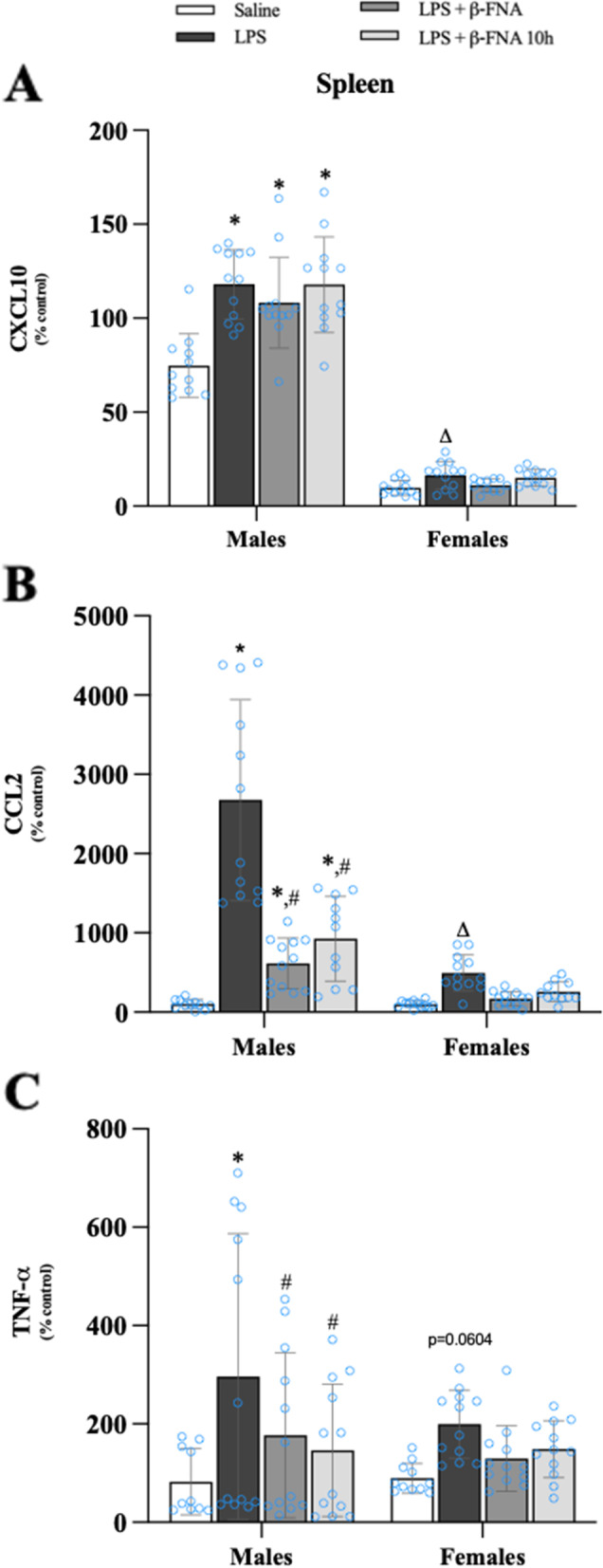
β-FNAs effect on LPS-induced CXCL10, CCL2, and TNF-α expression in the spleen of male and female C57BL/6J mice. Mice (*n* = 11–12/group) were injected (i.p.) with saline (control), LPS (0.83 mg/kg), LPS followed immediately by β-FNA treatment (50 mg/kg; i.p.; LPS + β-FNA), or LPS followed by β-FNA 10 h post-LPS (LPS + β-FNA 10 h). 24 h post-LPS, mice were terminated followed by tissue collection. Levels of CXCL10 (**A**), CCL2 (**B**), and TNF-α (**C**) of tissue homogenates were measured by ELISA. Data are reported as mean ± SEM. Two-way ANOVA indicated a significant main effect of treatment (*p* < 0.001), sex (*p* < 0.0001), and interaction (*p* < 0.001) on CXCL10 levels in the spleen. Two-way ANOVA determined CCL2 had a significant main effect of sex (*p* < 0.0001), treatment (*p* < 0.0001), and interaction (*p* < 0.0001). Two-way ANOVA determined TNF-α had a significant main effect of treatment (*p* < 0.003), but not sex (*p* = 0.25) or interaction (*p* = 0.54). Pairwise comparisons were assessed using a Fisher’s LSD test; * indicates *p* < 0.05 vs. saline group; # indicates *p* < 0.05 vs. LPS group; Δ indicates *p* < 0.05 vs. males LPS

The levels of CXCL10 in the spleen were significantly affected by treatment (F_3, 86_ = 12.71, *p* < 0.001) and sex (F_1, 86_ = 799.0, *p* < 0.0001); and there was a significant interaction (F_3, 86_ = 7.52, *p* < 0.001) (Fig. 9A). CXCL10 levels in the spleen were significantly greater in male LPS, LPS + β-FNA and LPS + β-FNA 10 h mice compared to male saline mice (all *p* < 0.0001), and there were no significant differences among the LPS-treated mice (*p* ≥ 0.1). There were no significant differences in spleen CXCL10 levels among the female groups (*p* ≥ 0.30), and LPS-induced upregulation of CXCL10 in the spleen was significantly less in female mice than in male mice (*p* < 0.0001).

There were significant main effects of treatment (F_3, 85_ = 37.05, *p* < 0.0001) and sex (F_1, 85_ = 59.16, *p* < 0.0001) on CCL2 expression in the spleen; and a significant interaction (F_3, 85_ = 19.93, *p* < 0.0001) (Fig. 9B). The levels of CCL2 were significantly increased in male LPS mice compared to male saline mice (*p* < 0.0001) and CCL2 expression was significantly decreased in male LPS + β-FNA and LPS + β-FNA 10 h mice compared to male LPS mice (*p* < 0.02 and *p* < 0.001, respectively). In females, there were no significant differences in spleen CCL2 levels of saline mice and any other group (*p* ≥ 0.06), and LPS-mediated upregulation of CCL2 in the spleen was significantly less in female mice than in male mice (*p* < 0.0001).

TNF-α levels in the spleen were significantly affected by treatment (F_3, 85_ = 5.30, *p* < 0.003), but no significant main effect of sex (F_1, 85_ = 1.37, *p* = 0.25) and no significant interaction (F_3, 85_ = 0.722, *p* = 0.54) (Fig. 9C). In males, LPS mice had higher levels of TNF-α levels in the spleen than did saline, LPS + β-FNA or LPS + β-FNA 10 h mice (*p* < 0.001, *p* < 0.04, and *p* < 0.01, respectively). TNF-α levels in the spleen of female mice were not significantly different among treatment groups (*p* ≥ 0.06).

We concluded from these findings that LPS-induced cytokine/chemokine expression in the spleen was most pronounced in male mice, and only CCL2 and TNF-α levels were inhibited by β-FNA.

### Effects of β-FNA on LPS-induced cytokine and chemokine levels in the liver

**Fig. 10:**
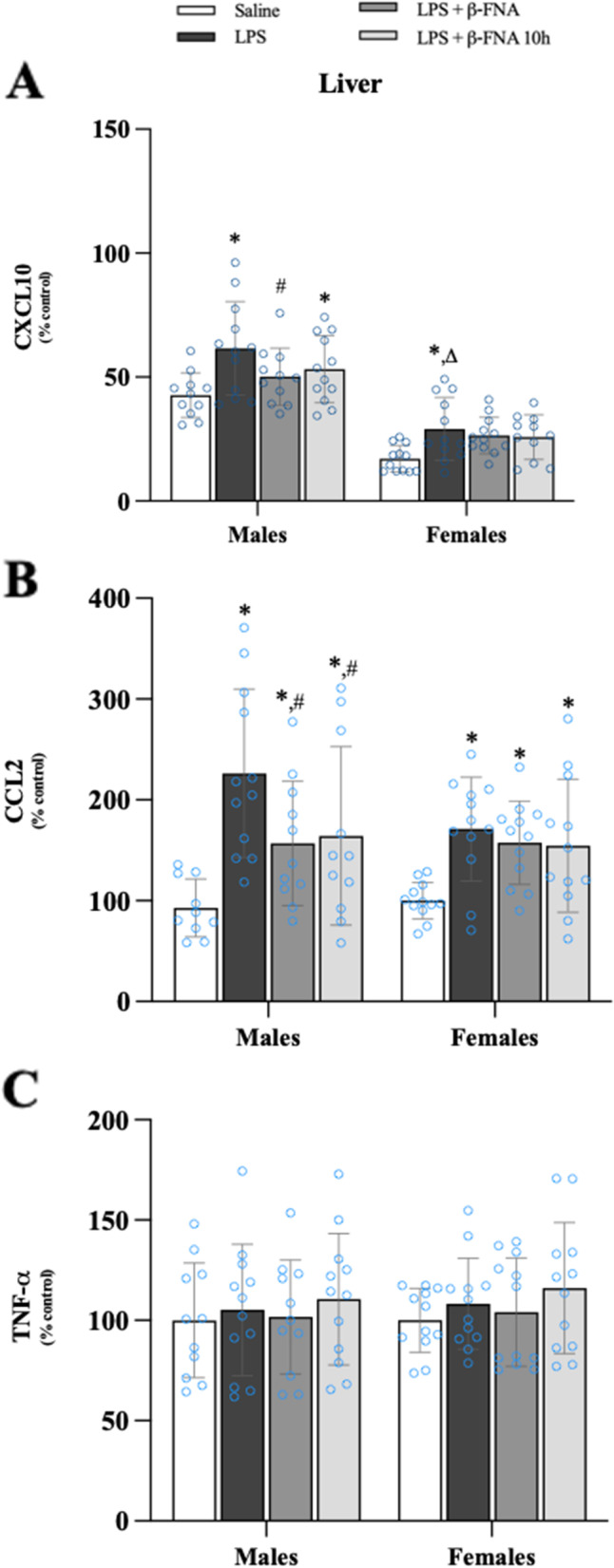
β-FNAs effect on LPS-induced CXCL10, CCL2, and TNF-α expression in the liver of male and female C57BL/6J mice. Mice (*n* = 11–12/group) were injected (i.p.) with saline (control), LPS (0.83 mg/kg), LPS followed immediately by β-FNA treatment (50 mg/kg; i.p.; LPS + β-FNA), or LPS followed by β-FNA 10 h post-LPS (LPS + β-FNA 10 h). 24 h post-LPS, mice were terminated followed by tissue collection. Levels of CXCL10 (**A**), CCL2 (**B**), and TNF-α (**C**) of tissue homogenates were measured by ELISA. Data are reported as mean ± SEM. Two-way ANOVA indicated a significant main effect of treatment (*p* < 0.001) and sex (*p* < 0.0001) on CXCL10 levels in the liver; but no significant effect on interaction (*p* = 0.61). Two-way ANOVA determined CCL2 had a significant main effect of treatment (*p* < 0.0001), but not of sex (*p* = 0.25) or interaction (*p* = 0.28). Two-way ANOVA determined TNF-α showed no main effects of sex (*p* = 0.63), treatment (*p* = 0.41), or interaction (*p* = 0.99). Pairwise comparisons were assessed using a Fisher’s LSD test; * indicates *p* < 0.05 vs. saline group; # indicates *p* < 0.05 vs. LPS group; Δ indicates *p* < 0.05 vs. males LPS

There were significant main effects of treatment (F_3, 85_ = 7.01, *p* < 0.001) and sex (F_1, 85_ = 127.3, *p* < 0.0001) on CXCL10 expression in the liver, but no significant interaction (F_3, 85_ = 0.617, *p* = 0.61) (Fig. 10A). In males, both LPS and LPS + β-FNA 10 h treated mice had significantly greater levels of CXCL10 in the liver than did male saline mice (*p* < 0.001 and *p* < 0.04, respectively), and the levels in LPS + β-FNA mice were significantly less than the levels in LPS mice (*p* < 0.03). CXCL10 levels in female LPS mice were significantly elevated compared to female saline mice (*p* < 0.02), yet similar to the levels in female LPS + β-FNA and LPS + β-FNA 10 h mice (*p* < 0.05). Also, LPS-induced liver CXCL10 expression was significantly lower in female mice than in male mice (*p* < 0.0001).

CCL2 levels in the liver were significantly affected by treatment (F_3, 84_ = 11.33, *p* < 0.0001) but not by sex (F_1, 84_ = 1.32, *p* = 0.25), nor was there a significant interaction of treatment and sex (F_3, 84_ = 1.31, *p* = 0.28) (Fig. 10B). Liver CCL2 levels were significantly increased in male LPS mice compared to male saline (*p* < 0.0001), LPS + β-FNA (*p* < 0.01), and LPS + β-FNA 10 h (*p* < 0.02) mice. In females, LPS (*p* < 0.01), LPS + β-FNA (*p* < 0.03), and LPS + β-FNA 10 h (*p* < 0.03) were all increased relative to saline mice. LPS-induced CCL2 levels were significantly lower in the liver of female mice compared to male mice (*p* < 0.03).

The levels of TNF-α in the liver were not significantly affected by treatment (F_3, 86_ = 0.982, *p* = 0.41) or sex (F_1, 86_ = 0.228, *p* = 0.63), nor was there a significant interaction (F_3, 86_ = 0.039, *p* = 0.99) (Fig. 10).

This experiment revealed that LPS-induced CXCL10 and CCL2 expression in the liver is inhibited by β-FNA (either immediate or 10 h post-LPS administration), but only in male mice.

### Effects of β-FNA on LPS-induced cytokine and chemokine levels in the intestines

**Fig. 11:**
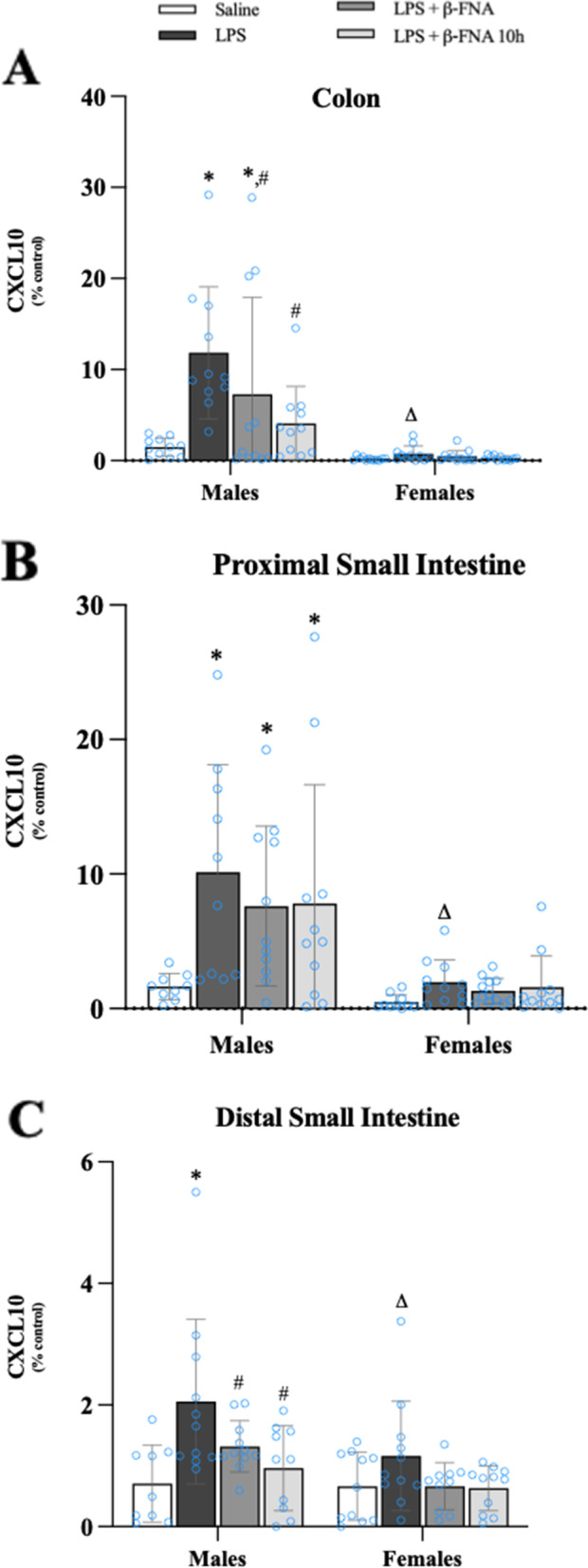
β-FNAs effect on LPS-induced CXCL10 expression in the colon, proximal small intestine (PSI), and distal small intestine (DSI) of male and female C57BL/6J mice. Mice (*n* = 11–12/group) were injected (i.p.) with saline (control), LPS (0.83 mg/kg), LPS followed immediately by β-FNA treatment (50 mg/kg; i.p.; LPS + β-FNA), or LPS followed by β-FNA 10 h post-LPS (LPS + β-FNA 10 h). 24 h post-LPS, mice were terminated followed by tissue collection. Levels of CXCL10 of the colon (**A**), PSI (**B**), and DSI (**C**) of tissue homogenates were measured by ELISA. Data are reported as mean ± SEM. Two-way ANOVA indicated a significant main effect of treatment (*p* < 0.01), sex (*p* < 0.0001), and interaction (*p* < 0.01) on CXCL10 levels in the colon. In the proximal small intestine two-way ANOVA determined CXCL10 showed main effects of sex (*p* < 0.0001) and treatment (*p* < 0.02), but not interaction (*p* = 0.14). In the distal small intestine two-way ANOVA determined CXCL10 showed main effects of sex (*p* < 0.005) and treatment (*p* < 0.001) but not an interaction (*p* = 0.28). Pairwise comparisons were assessed using a Fisher’s LSD test; * indicates *p* < 0.05 vs. saline group; # indicates *p* < 0.05 vs. LPS group; Δ indicates *p* < 0.05 vs. males LPS

Two-way ANOVA indicated significant main effects of treatment (F_3, 80_ = 5.31, *p* < 0.01) and sex (F_1, 80_ = 31.73, *p* < 0.0001) on CXCL10 levels in the colon, as well as a significant interaction (F_3, 80_ = 4.27, *p* < 0.01) (Fig. 11A). Pairwise comparisons showed a significant increase in CXCL10 levels in the colon of male LPS mice compared to male saline mice (*p* < 0.0001). Colon CXCL10 levels were significantly lower in male LPS + β-FNA (*p* < 0.03), and LPS + β-FNA 10 h (*p* < 0.001) mice compared to male LPS mice; with levels in LPS + β-FNA 10 h mice similar to the levels in saline mice (*p* = 0.20). In female mice, there were no significant differences among the treatment groups (*p* ≥ 0.79), and LPS-induced CXCL10 expression in the colon of female mice was significantly lower than in male mice (*p* < 0.0001).

In the proximal small intestine CXCL10 levels were significantly affected by treatment (F_3, 77_ = 3.77, *p* < 0.02) and sex (F_1, 77_ = 26.85, *p* < 0.0001), but there was not a significant interaction (F_3, 77_ = 1.89, *p* = 0.14) (Fig. 11B). In male mice CXCL10 levels in the proximal small intestine were significantly greater in LPS, LPS + β-FNA, and LPS + β-FNA 10 h compared to saline mice (*p* < 0.001, *p* < 0.01, and *p* < 0.01, respectively). There were no significant differences among the female groups.

(*p* ≥ 0.49), and LPS-induced CXCL10 expression in the proximal small intestine of female mice was significantly lower than in male mice (*p* < 0.0003).

CXCL10 levels in the distal small intestine were significantly impacted by main effects of treatment (F_3, 76_ = 6.67, *p* < 0.001) and sex (F_1, 76_ = 8.78, *p* < 0.005) but there was no significant interaction (F_3, 76_ = 1.30, *p* = 0.28) (Fig. 11C). Levels of CXCL10 in the distal small intestine were significantly higher in LPS male mice compared to male saline mice (*p* < 0.0001) and relative to male LPS + β-FNA (*p* < 0.03) and LPS + β-FNA 10 h (*p* < 0.002), both of which had levels similar to saline mice (*p* > 0.05). There were no significant differences in distal small intestine CXCL10 levels among the female groups (*p* ≥ 0.11), and LPS-induced CXCL10 expression in the distal small intestine of female mice was significantly lower than in male mice (*p* < 0.01).

**Fig. 12:**
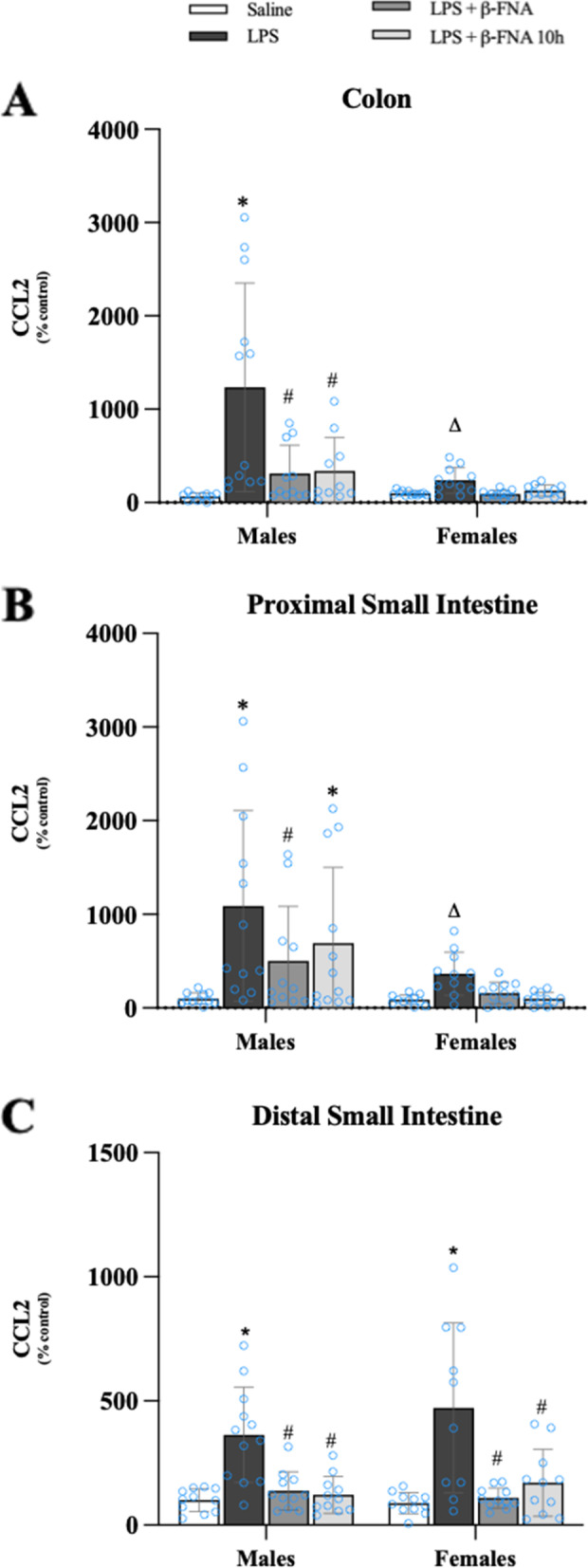
β-FNAs effect on LPS-induced CCL2 expression in the colon, proximal small intestine (PSI), and distal small intestine (DSI) of male and female C57BL/6J mice. Mice (*n* = 11–12/group) were injected (i.p.) with saline (control), LPS (0.83 mg/kg), LPS followed immediately by β-FNA treatment (50 mg/kg; i.p.; LPS + β-FNA), or LPS followed by β-FNA 10 h post-LPS (LPS + β-FNA 10 h). 24 h post-LPS, mice were terminated followed by tissue collection. Levels of CCL2 of the colon (**A**), PSI (**B**), and DSI (**C**) of tissue homogenates were measured by ELISA. Data are reported as mean ± SEM. Two-way ANOVA indicated a significant main effect of treatment (*p* < 0.0001), sex (*p* < 0.001), and an interaction (*p* < 0.002) on CCL2 levels in the colon. In the proximal small intestine two-way ANOVA determined CCL2 had a significant main effect of sex (*p* < 0.001) and treatment (*p* < 0.002) but not an interaction (*p* = 0.13). In the distal small intestine two-way ANOVA determined CCL2 had a significant main effect of treatment (*p* < 0.0001), but not sex (*p* = 0.37) or interaction (*p* = 0.42). Pairwise comparisons were assessed using a Fisher’s LSD test; * indicates *p* < 0.05 vs. saline group; # indicates *p* < 0.05 vs. LPS group; Δ indicates *p* < 0.05 vs. males LPS

In the colon, CCL2 levels were significantly affected by treatment (F_3, 80_ = 9.40, *p* < 0.0001) and sex (F_1, 80_ = 13.19, *p* < 0.001), and there was a significant interaction (F_3, 80_ = 5.66, *p* < 0.002) (Fig. 12A). Pairwise comparisons yielded a significant difference in CCL2 levels in the colon male LPS mice compared to saline, LPS + β-FNA, and LPS + β-FNA 10 h mice (all *p* < 0.0001), and levels in both β-FNA-treated groups were similar to saline males (*p* > 0.05). CCL2 levels in the colon of female mice were statistically similar among treatment groups (*p* ≥ 0.47), and the levels in LPS mice were lower than in male LPS mice (*p* < 0.0001).

The levels of CCL2 in the proximal small intestine were significantly affected by treatment (F_3, 82_ = 5.54, *p* < 0.002) and sex (F_1, 82_ = 14.07, *p* < 0.001), however there was not a significant interaction (F_3, 82_ = 1.97, *p* = 0.13) (Fig. 12B). CCL2 levels in male LPS mice were significantly greater than the levels in male saline (*p* < 0.0001) or LPS + β-FNA mice (*p* < 0.01); and the levels in LPS + β-FNA mice were not significantly different from the levels in saline mice (*p* = 0.08). CCL2 levels in the proximal small intestine of female mice were statistically similar among treatment groups (*p* ≥ 0.22), and the levels in LPS mice were lower than in male LPS mice (*p* < 0.002).

In the distal small intestine, CCL2 levels were significantly impacted by treatment (F_3, 80_ = 21.67, *p* < 0.0001), but not by sex (F_1, 80_ = 0.825, *p* = 0.37), nor was there a significant interaction (F_3, 80_ = 0.945, *p* = 0.42) (Fig. 12C). In male mice, distal small intestine CCL2 levels were significantly higher in LPS mice compared to saline (*p* < 0.0001), LPS + β-FNA (*p* < 0.001), and LPS + β-FNA 10 h mice (*p* < 0.001); and both β-FNA-treated groups were similar to saline mice (*p* ≥ 0.5). The pattern was similar in female mice as described for males. In females, CCL2 levels in the distal small intestine were significantly higher in LPS mice compared to saline, LPS + β-FNA, and LPS + β-FNA 10 h female mice (all *p* < 0.0001); and both β-FNA-treated groups were similar to saline mice (*p* ≥ 0.2).

**Fig. 13:**
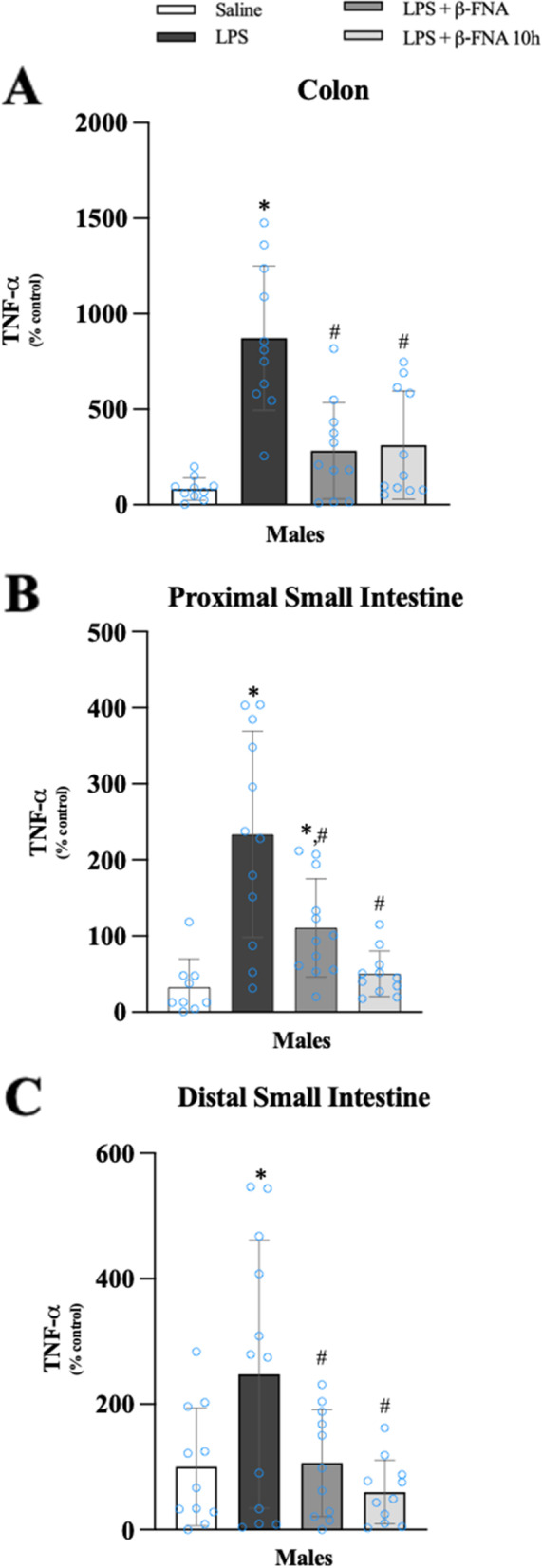
β-FNAs effect on LPS-induced TNF-α expression in the colon, proximal small intestine (PSI), and distal small intestine (DSI) of male C57BL/6J mice. Mice (*n* = 11–12/group) were injected (i.p.) with saline (control), LPS (0.83 mg/kg), LPS followed immediately by β-FNA treatment (50 mg/kg; i.p.; LPS + β-FNA), or LPS followed by β-FNA 10 h post-LPS (LPS + β-FNA 10 h). 24 h post-LPS, mice were terminated followed by tissue collection. Levels of TNF-α of the colon (**A**), PSI (**B**), and DSI (**C**) of tissue homogenates were measured by ELISA. Data are reported as mean ± SEM. One-way ANOVA indicated a significant main effect of treatment (*p* < 0.0001) on TNF-α levels in the colon, proximal small intestine (*p* < 0.0001), and distal small intestine (*p* < 0.01). Pairwise comparisons were assessed using a Fisher’s LSD test; * indicates *p* < 0.05 vs. saline group; # indicates *p* < 0.05 vs. LPS group; Δ indicates *p* < 0.05 vs. males LPS

After repeated attempts, it was determined that TNF-α levels in the intestines of female mice were below the level of detection; therefore, only data for males are reported. One-way ANOVA indicated a significant main effect of treatment (F_3, 39_ = 16.57, *p* < 0.0001) on colon TNF-α levels in male mice (Fig. 13A). Pairwise comparisons revealed that the levels of TNF-α in the colon were significantly greater in LPS mice compared to saline, LPS + β-FNA, and LPS + β-FNA 10 h males (all *p* < 0.0001); and both β-FNA-treated groups were similar to saline males (*p* ≥ 0.06).

The level of TNF-α in the proximal small intestine was significantly affected by treatment (F_3, 40_ = 13.73, *p* < 0.0001) (Fig. 13B). TNF-α levels in the proximal small intestine were significantly increased in LPS males compared to saline (*p* < 0.0001), LPS + β-FNA (*p* < 0.001), and LPS + β-FNA 10 h males (*p* < 0.0001); and the levels of TNF-α in LPS + β-FNA 10 h males were similar to the levels in saline males (*p* = 0.64).

One-way ANOVA indicated a significant effect of treatment (F_3, 41_ = 4.711, *p* < 0.01) on distal small intestine TNF-α levels in male mice (Fig. 13C). Pairwise comparisons revealed that the levels of TNF-α in the distal small intestine were significantly greater in LPS mice compared to saline (*p* < 0.01), LPS + β-FNA (*p* < 0.02), and LPS + β-FNA 10 h males (*p* < 0.002); and both β-FNA-treated groups were similar to saline males (*p* ≥ 0.4).

We concluded from these experiments that LPS-induced expression of CXCL10, CCL2, and TNF-α in the intestines was lower in females than in males. In the colon, β-FNA inhibits the induction of CXCL10 and CCL2 in a male-specific manner. In males, LPS-induced TNF-α is inhibited by β-FNA in colon, and small intestines.

### β-FNA effects on LPS-induced NFκB-p65 expression in the spleen and liver

**Fig. 14:**
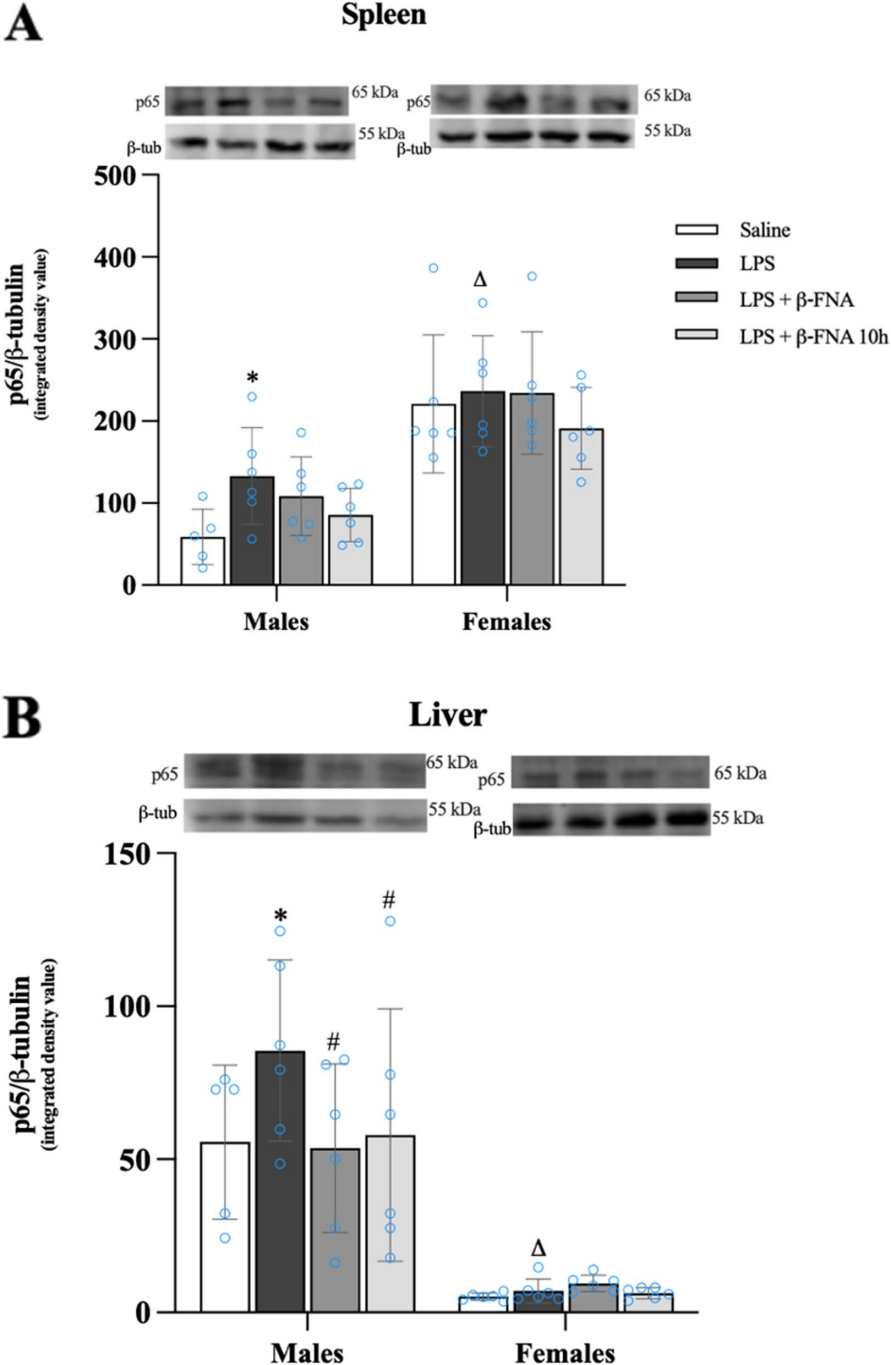
β-FNAs effect on LPS-induced NFκB-p65 expression in the spleen and liver of male and female C57BL/6J mice. Mice (*n* = 5–6/group) were injected (i.p.) with saline (control), LPS (0.83 mg/kg), LPS followed immediately by β-FNA treatment (50 mg/kg; i.p.; LPS + β-FNA), or LPS followed by β-FNA 10 h post-LPS (LPS + β-FNA 10 h). 24 h post-LPS, mice were terminated followed by tissue collection. Levels of NFκB-p65 of spleen **A** and liver **B** of tissue homogenates were measured by western blot analysis (representative western blots are shown above each area analyzed). Data are reported as mean ± SEM. Two-way ANOVA indicated a significant main effect of sex (*p* < 0.0001) on levels of NFκB-p65 in the spleen; but no significant effect of treatment (*p* = 0.16), nor a significant interaction (*p* = 0.62). In the liver two-way ANOVA determined levels of NFκB-p65 had a significant main effect of sex (*p* < 0.0001) but no significant effect of treatment (*p* = 0.28), nor a significant interaction (*p* = 0.26). Pairwise comparisons were assessed using a Fisher’s LSD test; * indicates *p* < 0.05 vs. saline group; # indicates *p* < 0.05 vs. LPS group; Δ indicates *p* < 0.05 vs. males LPS

Two-way ANOVA indicated that there was a significant main effect of sex (F_1, 39_ = 51.36 *p* < 0.0001) on NFκB-p65 levels in the spleen, yet there was no significant main effect of treatment (F_3, 39_ = 1.79 *p* = 0.16) nor was there a significant interaction (F_3, 39_ = 0.596, *p* = 0.62) (Fig. 14A, see next page). Pairwise comparisons revealed that male LPS mice expressed more NFκB-p65 in the spleen than did male saline mice (*p* < 0.05). β-FNA treated mice immediately or at 10 h were not significantly different from controls (*p* ≥ 0.10). In females, NFκB-p65 levels in the spleen were not significantly different among treatment groups, however, the levels in female LPS mice were significantly greater than in male LPS mice (*p* < 0.01).

In the liver, NFκB-p65 expression was significantly affected by sex (F_1, 39_ = 74.60 *p* < 0.0001), but there was no significant main effect of treatment (F_3, 39_ = 1.34 *p* = 0.28), nor was there a significant interaction (F_3, 39_ = 1.39, *p* = 0.26) (Fig. 14B). NFκB-p65 expression in the liver was significantly increased in male LPS mice relative to saline (*p* < 0.05), LPS + β-FNA (*p* < 0.02), and LPS + β-FNA 10 h mice (*p* < 0.05); and both β-FNA-treated groups were similar to saline mice (*p* ≥ 0.80). NFκB-p65 levels in the spleen of females were not significantly different among treatment groups (*p* ≥ 0.74); however, the levels in female LPS mice were significantly lower than in male LPS mice (*p* < 0.0001). Based on these findings, we concluded that NFκB-p65 expression is sex-dependent with females expressing more in the spleen; and males expressing more in the liver. Also, β-FNA inhibited LPS-induced NFκB-p65 expression in the liver of male mice.

## Discussion

Inflammation is involved in numerous neurological disorders, including anxiety, depression, and other mood disorders. It is also well established that diseases in peripheral tissues, such as the liver and intestines, involve inflammation. Furthermore, there is emerging evidence of the interplay between brain disorders and peripheral diseases, and inflammatory signaling is a primary link. Indeed, the literature is replete with reports of anxiety/depression exacerbating IBD and vice versa [18, 34, 35]. The literature seems to suggest that the prevalence of these diseases is greater in women than in men, but clearly, both men and women suffer from anxiety/depression and IBD [18, 36]. Studies in pre-clinical models of anxiety/depression and IBD have revealed differential severity of symptoms, disease progression, and response to treatment among males and females [37–40]. There are also reports of sex-dependent differences in inflammatory signaling [41–43]. Therefore, there remains much to learn about the role of sex in inflammatory diseases and the pharmacologic treatment of these disorders. In fact, it is expected that tailoring therapeutic strategies in a sex-specific manner will be effective for certain diseases, including anxiety, depression, and IBD [43–45].

In the present study, we assessed male and female mice in a pre-clinical model of LPS-induced inflammation to advance our understanding of the anti-inflammatory effects of β-FNA. We identified sex-dependent differences in behavioral deficits, inflammatory responses, and to some extent, the protective effects of β-FNA. In this section, we will discuss these key findings.

Consistent with our previous report, a single i.p. LPS injection resulted in anxiety-like behavior at 24 h [27]. Also, we observed minimal evidence of sickness behavior at 24 h post-LPS which is consistent with the findings of others that report sickness-like behavior at 6 h post-LPS is largely resolved by 24 h [46]. Importantly, we observed LPS-induced behavioral deficits in both male and female mice, which is particularly informative given that related studies reported in the literature have predominantly used male mice. However, more recently, there has been an increase in the number of investigations assessing both sexes. For instance, in 2022 Dockman et. al., [37] found that LPS (0.3 mg/kg, i.p.) resulted in more anxiety-like behaviors in females than in males. Whereas others administered a similar dose of LPS (0.3 mg/kg) via oral gavage and observed increased anxiety-like behavior in both male and female mice [42]. Interestingly, these researchers suggested that LPS-induced anxiety-like behavior may be regulated in part by sex-specific mechanisms [42]. We now report for the first time that β-FNA effectively reduces LPS-induced anxiety-like behavior. Follow-up studies with a more in-depth assessment of anxiety-like behavior, as well as other behaviors (e.g., depressive-like behavior), are warranted to gain a more robust appreciation of the effects of β-FNA.

Cytokines/chemokines are among the inflammatory mediators thought to contribute to the pathogenesis of anxiety/depression and mood disorders and have emerged as potential biomarkers of mood and anxiety disorders [47–49]. We previously reported that LPS-induced anxiety-like behavior in adult male mice positively correlated with IL-6 and CCL2 levels in the plasma and chemokine (CXCL10 and CCL2) expression in the brain [27]. Our present study supports these earlier findings of LPS-upregulated cytokine/chemokine expression in the brain. Importantly, we have extended our findings to include LPS-induced expression of inflammatory mediators in select brain regions of both male and female mice. We focused on the prefrontal cortex[50–52], hippocampus[53, 54], and cerebellum [55] as these regions are among those implicated in anxiety and mood disorders, particularly as associated with inflammation. We observed sex-dependent differences in cytokine/chemokine expression in the brain. For instance, LPS-induced CXCL10 expression in the prefrontal cortex and cerebellum/brain stem was more pronounced in males than in females. Whereas CCL2 expression was greater in the prefrontal cortex of females compared to males; yet CCL2 levels in the cerebellum/brain stem were lower in females relative to males. IL-6 was only significantly elevated in the cerebellum/brain stem of male mice. The sex-dependent differences are very interesting but not unexpected given the considerable evidence of sexual dimorphism in neuroimmune activation, including differences in microglia activation, cytokine expression, and immune-related receptors [43, 56, 57].

While we know that β-FNA can inhibit LPS-induced CXCL10 and CCL2 expression in the brain, we now show for the first time that β-FNA inhibited CXCL10 across brain regions, but only in male mice, whereas CCL2 was inhibited only by β-FNA in the cerebellum/brain stem and again only in male mice. LPS-induced IL-6 expression was only elevated in the cerebellum/brain stem and only in male mice; and downregulated by β-FNA. Sex-dependent differences in drug effects are not novel per se, particularly in terms of anti-depressants and anti-inflammatory agents [40, 58]. For example, there are sex differences in the effectiveness and tolerability of anti-TNF agents [59, 60]. In another example, glucosamine inhibited post-ischemic inflammation more effectively in male mice [61]. However, it is certainly noteworthy that the anti-inflammatory actions of β-FNA are sex-dependent.

The β-FNA-mediated anti-inflammatory effects in the hippocampus and prefrontal cortex are promising in terms of novel therapeutic approaches for anxiety/depression, given the importance of these regions in the neuropathogenesis of anxiety/depression [20–22]. For instance, inhibition of neuroinflammation in the hippocampus and prefrontal cortex was associated with reduced anxiety/depressive-like behavior [62–65]. However, the cerebellum is generally less studied in the context of anxiety/depression, but increasing evidence implicates this brain region in anxiety/depression and is thus a potential therapeutic target [66–68].

Based on the paucity of information in the literature, it is clear that the field is in the very early stages of understanding of β-FNA’s anti-inflammatory and neuroprotective effects *in vivo.* The first report on the anti-inflammatory effects of β-FNA came out of our lab [30]. The initial study, and subsequent investigations by our group used an *in vitro* approach to demonstrate the anti-inflammatory effects of β-FNA in human astroglial cells [29]. The first report of β-FNA being anti-inflammatory in mice came several years later in which a high dose of LPS (5 mg/kg; i.p.), a relatively lower dose of β-FNA (28 mg/kg; i.p.) were assessed [28]. We determined that β-FNA inhibited LPS-induced CXCL10 expression in the brain (but not in the plasma) and failed to prevent sickness-like behavior at this early time point. Thereafter, we found that β-FNA (12.5 – 50 mg/kg; i.p.) inhibited LPS (0.083 mg/kg, i.p.)-induced CXCL10 and CCL2 expression in the brain at 24 h post-treatment when administered concurrently with LPS [27]. Outside of our lab, there is one other report on the anti-inflammatory of β-FNA *in vivo* in which β-FNA (intraventricular infusion) protected against cerebral ischemia/reperfusion injury and reduced expression of several inflammatory factors in rats [69].

It is not only crucial to understand the effects of β-FNA on neuroinflammatory signaling, but also the impact on inflammatory mediators in peripheral tissues. For instance, intestinal inflammation exacerbates anxiety/depression symptoms, which has been widely reported in the context of IBD [18, 34, 35]. Others found that CXCL10 expression is increased in colonic mucosa of patients with active IBD [70]. Cluny et al., [71] recently reported that liver and gut inflammation leads to neuroinflammation as indicated by increased TNF, IL-1β, and CCL2 levels in the brain. These findings are consistent with the LPS-induced neuroinflammation coupled with inflammatory cytokine/chemokine expression in the spleen, liver, and intestines. We are particularly intrigued by the pronounced anti-inflammatory effects of β-FNA on LPS-induced CXCL10, CCL2, and TNF-α expression in the intestines and the fact that these inhibitory effects were largely limited to the male mice. These results are promising, considering the reported benefits of anti-inflammatory/immunomodulatory agents in treating IBD, and associated anxiety and depression were recently reported [72]. These investigators revealed that anti-TNF therapy combined with immunomodulatory therapy (azathioprine, 6-mercaptopurine, or methotrexate) reduced both disease activity and symptoms of anxiety/depression in patients with IBD. Thus, we are interested in further exploring the anti-inhibitory and protective effects of β-FNA in a pre-clinical model of IBD. The transcription factor NF-κB is instrumental in the upregulation of inflammatory mediators, including the cytokines/chemokines assessed in this study. Nuclear factor kappa B p65 (NF-κB-p65) is one of the five components that form the NF-κB/RE1 family [73]. Homodimers or heterodimers remain as inactive complexes with inhibitor of nuclear factor κB (IκB) inhibitory proteins in resting cells; upon phosphorylation, the NF-κB subunit p65 translocates into the nucleus, and binds promoters to induce the expression of pro-inflammatory protein genes [74]. The elevation of p65 in the cell has been linked to neurodegenerative diseases, rheumatoid arthritis, and inflammatory bowel disease [73, 75]. Interestingly, it was very recently demonstrated in a pre-clinical experimental model, that the CCL2/NF-κB signaling pathway is implicated in the pathogenesis of colitis and suggested as a therapeutic target [76].

Thus, β-FNA-mediated inhibition of CCL2 and NF-κB expression in both the brain and intestines is quite compelling. We also found that β-FNA inhibited LPS-induced CCL2 and TNF-α expression in the spleen; while in the liver, CXCL10 and CCL2 were inhibited. Furthermore, NF-κB p65 expression in the spleen and liver was inhibited by β-FNA. Interestingly, β-FNA inhibited these factors seemingly in a male-specific manner, which may prove to be useful information in terms of targeting therapeutic strategies in male patients. However, follow-up studies are required to determine whether this holds true.

In contrast to the spleen and liver, β-FNA downregulated LPS-induced NF-κB p65 expression in the brain of male and female mice, and the hippocampus seemed to be most responsive. It remains unclear why there are temporal differences in the effects of β-FNA on NF-κB p65 expression between male and female mice. While these findings are promising in terms of showing that the NF-κB pathway is impacted by β-FNA, they are limited in part by the fact that we did not measure NF-κB activation per se. However, we have determined *in vitro* that β-FNA does in fact inhibit NF-κB activation and subsequent production of chemokines in astrocytes [25, 30, 77]. Further investigation is warranted in order to gain further mechanistic insight into β-FNA effects *in vivo*.

## Conclusion

In summary, we have advanced our understanding of the anti-inflammatory actions of β-FNA in a pre-clinical model of LPS-induced inflammation. The inclusion of male and female mice in the study enabled us to identify sex-dependent differences in inflammatory signaling and in β-FNA-mediated effects. Inhibition of both neuroinflammation and intestinal inflammation, as well as reduced anxiety-like behavior, by β-FNA is promising in terms of potential therapeutic options for anxiety/depression and/or IBD. The sex-dependent effects of β-FNA require further investigation but may be particularly translational in developing sex-specific treatments. Subsequent investigations to further define inflammatory signaling events that are modulated by β-FNA and obtain additional mechanistic insights will be beneficial. Future studies on repeated β-FNA administration are warranted in this model as well as other models of inflammation (e.g., IBD) and in combination with other pharmacologic agents such as secreted serotonin reuptake inhibitors.

## Materials and methods

### Animals

USDA-approved facilities at Oklahoma State University-Center for Health Sciences (OSU-CHS) were used to house ninety-six seven-week-old male (*n* = 48) and female (*n* = 48) C57BL/6J mice (Jackson Laboratories, Bar Harbor, ME). Each cage (10 cm × 17 cm × 28 cm) housed three mice and was equipped with pine chip bedding, ad libitum access to food and water, and cardboard tubes for environmental stimulus. The room was placed on a 12:12 light: dark cycle with a steady ambient temperature of 21 °C. Before the initiation of experiments, the mice were afforded a 7-day acclimation period. OSU-CHS Institutional Animal Care and Use Committee approved all experimental processes and animal manipulations, and all mice were monitored daily.

### Experimental protocol

Mice (*n* = 11–12 per group) were injected with an intraperitoneal (i.p.) dose of LPS (0.83 mg/kg dissolved in saline; *Escherichia coli* O55:B5; Sigma) as previously described [27]. This dose of LPS was previously documented to effectively induce behavioral deficits, such as sickness behaviors and anxiety-like behavior, and neuroinflammation in mice [5, 27, 78, 79]. β-FNA (50 mg/kg, dissolved in saline, i.p.; National Institute on Drug Abuse reagent supply program) was administered immediately or 10 h post-LPS; and control mice received vehicle (200 µl saline). The β-FNA dose used in this study was derived from our previous work [27, 80]

### Behavioral measures

Sickness and anxiety-like behavior were assessed 24 h post-LPS administration using the open-field test (OFT) and elevated plus maze (EPM), respectively, as previously described [5, 27, 78, 79]. For the OFT, the mouse was placed in the center of the arena (40 cm × 40 cm) then monitored (and recorded) for 10 min using Ethovision Software. Distance moved (cm) was used as a dependent measure, where reduced distance moved (compared to controls) was indicative of sickness behavior [5, 78, 79]. The EPM was composed of two closed arms (25 cm × 5 cm × 16 cm) and two open arms (25 cm × 5 cm × 0.5 cm), and a center area (5 cm × 5 cm × 0.5 cm) [81]. Each mouse was placed in the center of the maze, then monitored and recorded for 5 min using Ethovision Software. Reduced time (sec.) spent in the open arms (compared to control mice) was interpreted as anxiety-like behavior [81].

### Tissue and total protein levels collection

Immediately following behavioral analyses, mice were euthanized by CO_2_ inhalation and subsequent decapitation. The following tissues were collected: trunk blood, brain, spleen, liver, colon, and small intestines. All tissue samples were collected into ice-cold tubes and maintained on ice. Plasma was collected immediately by centrifugation (17,000 × g, 15 min., 4 °C). Plasma and tissues were then stored at − 80 °C. In select experiments, the hippocampus, prefrontal cortex, and cerebellum/brain stem were dissected from the whole brain and stored at − 80 °C. Tissue preparation for biochemical assays was done as previously described [82]. Briefly, tissues were homogenized with a Sonic Dismembrator Model 100 (Fisher Scientific) in ice-cold triple detergent lysis buffer containing HALT Protease/Phosphatase Inhibitor Cocktail (Thermo Fisher Scientific) [83]. The aqueous phase was collected after centrifugation (20,000 × g, 20 min, 4 °C). Total protein levels were determined using the bicinchoninic acid (BCA) protein assays as previously described [82].

### Cytokine/chemokine levels

IL-1β, IL-6, TNF-α, CXCL10, and CCL2 in plasma and tissue homogenates were quantified using dual-antibody solid-phase immunoassays (ELISA Development Kit, Peprotech) according to the manufacturer's instructions. A BIOTEK Synergy 2 spectrophotometer was used to read the absorbance at 450 nm using a microplate reader and imager software Prism 9.

### NF-κB-p65 levels

The expression of NF-κB-p65 in tissue homogenates was determined by western blot analysis. Protein (100 µg) was separated by 7.5% SDS polyacrylamide gel electrophoresis and transferred to a PVDF membrane as previously described [77, 84]. The membrane was incubated overnight at 4 °C in an NF-κB-p65 1° antibody (1:1000; Cat# 4764S, Cell Signaling Technology). The membrane was then washed six times in Tris-buffered saline with 0.1% Tween (TBST); then incubated in 2° antibody [Goat-anti-Rabbit IgG (1:10,000), Cat# 926–32212; Li-Cor) for 2 h at room temperature. Membranes were washed, then images were obtained using a Licor-CLX Odyssey. For normalization, membranes were stripped of antibody with stripping buffer (Cat# 21059; Thermo Fisher Scientific), then re-probed with β-tubulin (1:1000, Cat# 2146S; Cell Signaling Technology), labeled with 2° antibody, and imaged as above. NIH Image J was used for the relative quantification of protein signals.

### Statistical analysis

A two-way ANOVA (treatment × sex) and a one-way ANOVA were used to analyze data, and Fisher's LSD was used for pairwise comparisons. Data are presented as mean ± SEM, and *p*-values < 0.05 are considered statistically significant. ${\mathrm{Prism}}^{\mathrm{TM}}$ Version 9 software (GraphPad Inc, San Diego, CA) was used for data analysis and figure preparation.

## Data Availability

Data can be made available upon reasonable request.

## Abbreviations

β-FNA: Beta-funaltrexamine
MOR: Mu-opioid receptor
LPS: Lipopolysaccharide
IP-10 (CXCL10): Interferon γ-induced-protein 10
MCP-1 (CCL2): Monocyte-chemotactic-protein 1
IL-6: Interleukin-6
IL-1β: Interleukin-1β
TNF-α: Tumor necrosis factor-alpha
NF-κB: Nuclear factor-kappa B
OFT: Open field test
EPM: Elevated plus maze
IBD: Inflammatory bowel disease
CD: Crohn's disease
